# Ecological Factors Mediate Immunity and Parasitic Co-Infection in Sea Fan Octocorals

**DOI:** 10.3389/fimmu.2020.608066

**Published:** 2021-01-11

**Authors:** Allison M. Tracy, Ernesto Weil, Colleen A. Burge

**Affiliations:** ^1^ Department of Ecology and Evolutionary Biology, Cornell University, Ithaca, NY, United States; ^2^ Department of Marine Sciences, University of Puerto Rico, Mayagüez, PR, United States; ^3^ Institute of Marine and Environmental Technology, University of Maryland Baltimore County, Baltimore, MD, United States

**Keywords:** ecological immunity, *Gorgonia ventalina*, co-infection, host demography, octocoral

## Abstract

The interplay among environment, demography, and host-parasite interactions is a challenging frontier. In the ocean, fundamental changes are occurring due to anthropogenic pressures, including increased disease outbreaks on coral reefs. These outbreaks include multiple parasites, calling into question how host immunity functions in this complex milieu. Our work investigates the interplay of factors influencing co-infection in the Caribbean sea fan octocoral, *Gorgonia ventalina*, using metrics of the innate immune response: cellular immunity and expression of candidate immune genes. We used existing copepod infections and live pathogen inoculation with the *Aspergillus sydowii* fungus, detecting increased expression of the immune recognition gene Tachylectin 5A (T5A) in response to both parasites. Cellular immunity increased by 8.16% in copepod infections compared to controls and single *Aspergillus* infections. We also detected activation of cellular immunity in reef populations, with a 13.6% increase during copepod infections. Cellular immunity was similar in the field and in the lab, increasing with copepod infections and not the fungus. Amoebocyte density and the expression of T5A and a matrix metalloproteinase (MMP) gene were also positively correlated across all treatments and colonies, irrespective of parasitic infection. We then assessed the scaling of immune metrics to population-level disease patterns and found random co-occurrence of copepods and fungus across 15 reefs in Puerto Rico. The results suggest immune activation by parasites may not alter parasite co-occurrence if factors other than immunity prevail in structuring parasite infection. We assessed non-immune factors in the field and found that sea fan colony size predicted infection by the copepod parasite. Moreover, the effect of infection on immunity was small relative to that of site differences and live coral cover, and similar to the effect of reproductive status. While additional immune data would shed light on the extent of this pattern, ecological factors may play a larger role than immunity in controlling parasite patterns in the wild. Parsing the effects of immunity and ecological factors in octocoral co-infection shows how disease depends on more than one host and one parasite and explores the application of co-infection research to a colonial marine organism.

## Introduction

Rapid and widespread shifts in the environment have altered the impact of disease on wildlife populations at a global scale. Changes in the environment, such as increased warming, can increase disease risk by altering parasite physiology and compromising host immune defenses (1, 2). At the same time, host demography and life history traits also govern immunity and disease risk in the wild (3, 4). Despite the catastrophic consequences of shifting disease risk, the interplay between the environment, demography, and host-parasite interactions is poorly understood. Further integration of ecological immunology and disease ecology is necessary to elucidate how host immunity functions in nature and scales up to affect disease in multi-parasite systems.

The role of ecological factors in modulating immunity is especially critical for environmentally sensitive organisms. In many of these organisms, including amphibians, shellfish, and corals, compromised immunity can facilitate environmentally-mediated disease outbreaks (5–7). The uptick in coral disease with warming is one of the clearest examples (8–10). Temperature is the most prominent driver, but other conditions, such as pollution, also alter coral disease risk (11–13).

The functioning of the coral immune repertoire in a changing environment is of special interest because cnidarians are among the most ancient invertebrates. Key components of cnidarian innate immune systems include recognition, signaling, effector responses and wound healing that are mediated by both cellular and humoral factors and lead to active engulfment, melanin barriers, and antimicrobial peptides (14). Studies at the vanguard of research on the coral immune system have identified genes that shift with artificial pathogen elicitors (15, 16), ecologically relevant pathogens (17), preexisting disease lesions (18–20), and bleaching events (21, 22). Gene expression studies reveal the pathways that corals activate during pathogenic attacks and identify candidate genes for future research. They also confirm that the environment can alter coral immunity in the absence of a parasite (21, 23, 24). Immune responses in the presence of stressors other than parasites are common in invertebrates and have also been observed for coral cellular immunity (13, 25, 26). Despite many recent advances in coral immunology, no studies have explored coral immune profiles with more than one parasite. There is rising interest in incorporating co-infection into studies of eco-immunology to understand patterns of disease in nature (27, 25).

Recent studies have greatly advanced research on the complex interplay between host- and population-level factors during co-infection. They have elucidated underlying mechanisms of parasite interactions during co-infection, such as parasite resource use, host immunity, and direct killing of one species of parasite by another. Furthermore, they have demonstrated how these mechanisms scale up to affect disease dynamics at the population level (27, 28, 29). One overarching finding is that patterns of parasite co-occurrence cannot alone reveal the nature of the interaction between parasites. Factors including host mortality, parasite resource use, and host immune responses together shape co-occurrence patterns, meaning that parasites facilitating each other immunologically may not positively co-occur (30–32). Similarly, the negative pattern expected from immune-mediated antagonism between parasites combined with the positive force of common resource use could equilibrate as random co-occurrence, as suggested for an amphibian co-infected with two trematodes (33). Studying co-infection in corals contributes an important perspective on innate immunity, which is shared by all metazoans, and expands on a relatively small body of work on co-infection in marine organisms. In particular, the colonial life history of corals adds an interesting dimension to co-infection theory because there are no clear expectations for interactions between parasites inhabiting different tissues or polyps (i.e., individuals) within a colonial organism. Parasite resource use and host immunity are two of the major factors regulating co-infection within hosts, as parasites can compete for resources and help or harm each other through host immunity. The degree to which this theoretical basis applies to corals is unclear because infections in neighboring coral polyps could conceivably be isolated from each other with respect to both resources use and immune responses. Our investigation of immunity, disease, environment and demography in octocorals is a pioneering effort to address pressing questions on co-infection in an underexplored immunological context.

Disentangling the role of co-infection in coral disease outbreaks is critical, as infections with multiple parasites are common and can exacerbate the toll of epizootics (28). Disease-induced declines in corals threaten not only the hosts, but also the extensive marine biodiversity that relies on these ecosystem engineers. The Caribbean sea fan octocoral, *Gorgonia ventalina*, is a tractable host in which to explore the mechanisms and consequences of coral co-infection because sea fans have several known parasites, whereas disease etiology is lacking for most other coral diseases (34, 35). Defined immune responses in sea fans support an eco-immunological approach to sea fan disease, which can help understand causes and offer solutions (12, 36). Sea fans are also important habitat formers in their own right, and may have an oversized role in building reefs in the Anthropocene (37).

**Figure 1:**
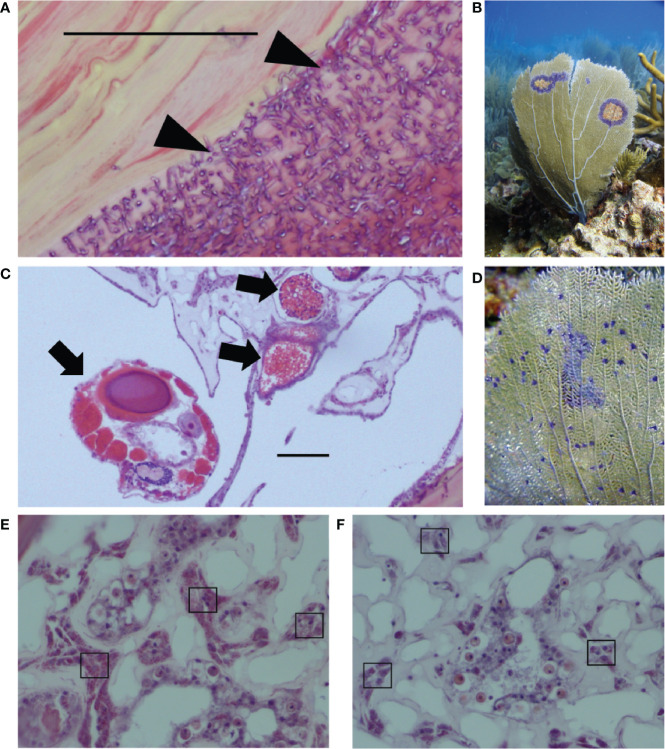
Two of the major sea fan parasites in the wild are **(A)** fungal hyphae that infect the gorgonin skeleton (see triangles), leading to purple halos shown in the field in **(B)**; and **(C)** the copepod parasite infecting the mesoglea (see arrows), linked to multi-focal purple spots shown in the field in **(D)**. Images from field samples that are representative of both field and lab samples show examples of **(E)** high amoebocyte density and **(F)** low amoebocyte density, with examples of dark pink amoebocytes in boxes. Scale bars = 0.50 μm. Photo credit: EW.

This study characterizes previously unknown rates of co-infection and explores drivers of variation in coral immunity in the context of co-infection, focusing on two known parasites of *G. ventalina*. The first parasite, the *Aspergillus* fungus, caused a damaging epizootic in the early 2000s (38). Since then, studies have shown that multiple fungal species can cause disease, that *A. sydowii* can be present without external signs of disease, and that *Aspergillus* suppresses reproduction (39, 40). Irrespective of their identity, fungal infections damage the gorgonin skeletal axis of *G. ventalina* (**Figure 1A**). The second parasite is a copepod that infects the connective tissue, or mesoglea, and is linked to an emerging disease called “multi-focal purple spots” (MFPS), characterized by small purple spots of 1–3 mm (35, 41–43). Copepod infections compromise reproduction in sea fans, but did not induce a cellular immune response in a previous field survey (44, Tracy et al., unpublished data; **Figure 1B**).

The immune capabilities of *G. ventalina* are unusually well characterized due to the *Aspergillus* outbreak in the early 2000s (38), which revealed the central role of cellular immunity, in the form of granular amoebocytes, and biochemical effector responses (25, 45, 46). More recently, a sea fan transcriptome study highlighted immune recognition and effector genes that respond to a labyrinthulid parasite (17), which allowed us to target candidate immune genes in this study. Multiple resources for measuring immunity are uncommon and highly valuable for non-model species, especially outside mammals (36). Previous studies on *G. ventalina* have shown that temperature, water depth, pollutants and sea fan colony size influence immunity, providing a tractable system to study the interplay of immunity with environment and demography amidst co-infection (13, 25; 44, 47).

The aim of this study was to explore the role of ecological factors in immunity and co-infection in a wild population of *G. ventalina*. The first objective was to test whether sea fan immune responses depend on which parasite is present and whether there is single or co-infection. We evaluated this question in a laboratory setting by measuring cellular immunity and gene expression in a 2x2x2 factorial design with pre-existing copepod infections, live pathogen inoculations with *Aspergillus* cultures, and 2 time points. We also employed field surveys of the natural population to complement laboratory results. The second objective was to test how immunity and disease in the field depend on infection, environmental factors and host demographic factors. The third objective was to characterize parasite co-occurrence in the field. We assessed patterns in the field with the second and third objectives to lay the groundwork for future mechanistic approaches to studying how within-host interactions scale to population-level patterns. The results illustrate how immunity contributes to co-infection dynamics in nature in light of environmental conditions and demographics of natural host populations.

## Materials and Methods

### Immune Profiling of Sea Fans With Single and Co-Infections in Laboratory Exposures

#### Laboratory Design

Host immune metrics during infection with the *Aspergillus sydowii* fungus and the copepod were characterized using a factorial laboratory experiment at the Isla Magueyes Marine Lab of the University of Puerto Rico in La Parguera. We collected sea fan samples (Permit # O-VS-PVS15-SJ-00671-24042014) for the experiment on 29 June 2015 from Laurel Patch (17° 56.480 N and 67° 04.484 W), as this site had sufficiently high disease prevalence of MFPS. For each colony (N=10 colonies), we used gloves and titanium scissors to collect five samples with MFPS and five asymptomatic samples (N=10 samples/colony). As in most wild systems, acquiring disease-free samples is usually not possible in *G. ventalina*, but we were able to use external signs of disease as a guide. We used four of the five samples of each type from each colony in the treatments (N=80 samples, 4 treatments x 2 time points x 10 colonies), but the fifth tissue sample of each type was used to assess whether there were heavy preexisting infections that justified omitting certain colonies (N=20, 2 x 10 colonies). All samples were stored in seawater in Ziplocs at ambient temperature for transport to the lab. They were trimmed to 5x5 cm, suspended on fishing wire and acclimated for 48 h in the final treatment aquaria (17).

The 20 plastic aquaria were randomly assigned to 20 positions across two tables. Then, four sea fan samples per treatment from different colonies were randomly selected for each aquarium, with five tanks for each of the four treatments. Each tank included two samples for each of the two sampling time points, so as not to confound tank with sampling time. Each side of each table was lit by a 120-volt light (Aquatic Life LLC Model #LF4-48), set to a daily cycle of actinic light from 7:00–19:00 and white light from 8:00–18:00 to replicate natural conditions. We filled each aquarium daily with 22 L of sand-filtered seawater pumped into the flow-through seawater system of the marine station from a nearby channel. Each tank received aeration from air pumps and dispersion stones to maintain oxygen levels and water movement. Temperature (27–29°C) and salinity (38 ppt) in the aquaria represented ambient conditions in the field.

The laboratory design (**Appendix S1: Figure S1**) included four parasite treatments (existing copepod infections, *Aspergillus sydowii* inoculations, both, or neither), each with 20 samples for 10 colonies to enable sampling at 6 h (N=10 x 4 treatments) and 48 h (N=10 x 4 treatments). The experiment was clonally replicated with a sample from each colony for every treatment and time point (N=8 samples/colony). We used preexisting copepod infections based on MFPS because the copepod parasite is not in culture. Our previous work shows most purple spots are indicative of copepod infections (44). Successive years of field surveys also indicate that copepod infections are long-lived (13), suggesting they may often be the earlier infection.

We measured amoebocyte density and the expression of candidate immune genes at the two time points. We selected the 6-h time point to compare our results to previous studies of immune responses to pathogens and environmental stress (23, 48). Previous studies in sea fans did not have sufficiently early time points to measure this critical, early immune response. We chose the 48-h time point based on previous studies of sea fan amoebocyte responses and gene expression that measured responses to pathogens at 24- and 48-h post infection (17, 49).

#### Laboratory Inoculations

Sea fans were inoculated on 1 July 2015 in the center of each piece with fungal hyphae from *Aspergillus sydowii* strain GSS7A (see 46 for inoculum prep) in SSW or a SSW control. Half of the colonies were processed at 6 h post-injection on 1 July 2015 and the other half were processed at 48 h on 3 July 2015. Each piece was divided into 4 pieces, each with a quarter of the area surrounding the injection point. One sample and two back-ups for real time quantitative-PCR (RT-qPCR) were flash frozen in liquid nitrogen and stored at -80°C. The 4th piece was fixed in 10% formalin for 24 h, then switched into 70% ethanol for histology (see *Field and Laboratory Analyses*).

#### Selection of Candidate Genes

We selected three candidate immune genes to represent three distinct arms of the octocoral immune system in the laboratory experiment: recognition, effector mechanisms, and wound healing (**Appendix S1: Table S1**). Candidate immune genes are submitted to GenBank as accessions numbers XX-XX (submission pending). The first candidate gene, Tachylectin 5A (T5A), is a lectin used in pathogen recognition that was differentially expressed in response to the labyrinthulid parasite in *G. ventalina* (14, 17, 50). Other tachylectin forms are present in other coral species (14), and lectins of various kinds have also been differentially expressed in studies of disease lesions and gene expression (18) and artificial pathogen elicitors and protein expression (48) in other coral species. The second candidate gene, inhibitor of NF-κB (IκB) cactus, was selected from a contig in the sea fan transcriptome based on its annotated function (from *Drosophila melanogaster*) as a gene that inhibits the NF-κB transcription factor, an important activator in the Toll pathway involved in activating melanin synthesis and other innate immune defenses (14, 51). NF-κB homologs exist in corals and acidification, thermal stress, and nutrient pollution alter coral NF-κB and IκB expression (24, 52–54). Differential expression was observed in response to an artificial pathogen elicitor in the reef-builder *Orbicella faveolata* (16), and bacterial challenge in *Pseudodiploria strigosa* (55). The third candidate gene, matrix metalloproteinase (MMP), was differentially expressed in the *G. ventalina* response to the labyrinthulid (17). Matrix metalloproteinases have several functions, including in melanosomes and wound healing in other cnidarians (56). We used elongation factor 1 (EF1) as a reference gene based on its stability in previous analyses of sea fan gene expression (17).

#### Gene Expression With RT-qPCR

RNA was extracted from a 2x2cm tissue sample for each of the 80 *G. ventalina* samples from the laboratory experiment. We used a modified phenol-chloroform RNAEasy protocol described in Burge et al. (17). Following RNA exactions, DNA was removed using the Turbo DNA-free treatment according to the manufacturer’s instructions (Ambion Inc, The Life Technologies™, Grand Island, New York). RNA concentrations were measured using a Thermo Scientific Nanodrop ND1000 (see **Appendix S1: Table S2**). cDNA was created using 1 µg of RNA using the GoScript Reverse Transcription System (Promega) following the manufacturer’s protocol, and stored at -20C.

Quantitative PCR (qPCR) was employed using three candidate genes (**Appendix S1: Table S2**). qPCR reactions (20 µl) were performed in duplicate in 96-well plates (Bio-Rad Hard-Shell^®^ 96-Well PCR Plates) including 10 µl of Fast SYBR^(R)^ Green Master Mix (Applied Biosystems, The Life Technologies Corporation™, Grand Island New York), 5 µg BSA, and 400 nM of each primer. Template (2 µl) added to reactions were sea fan cDNA or DNAsed RNA [to ensure no DNA contamination or no-template controls (N=2 per plate)]. All qPCR reactions were run on a Bio-Rad CFX Connect 96 Real-Time PCR Detection system with the following reaction cycle: 95°C for 20 s, followed by 40 cycles of 95°C for 3 s 60°C for 30 s, and finally a melt curve analysis at 65°C for 5 s and 94°C for 5 s to confirm amplification of a single product. We calculated fold change in gene expression relative to the reference gene, EF1. Real-time PCR miner was used to calculate primer efficiencies and cycle threshold values (**Appendix S1: Table S3**), which were incorporated into the following equation to calculate relative expression: 1/(1 + Efficiency)^Cq (57).

### Parasite Co-Occurrence and Sea Fan Immunity in the Field

Field surveys were conducted from 10 June to 3 July 2015 at 15 field sites in the La Parguera Natural Reserve and Guánica, Puerto Rico (**Appendix S1: Figure S2**, **Table S4**), as described in Tracy et al. (44). In brief, all sea fan colonies at 3 haphazardly-deployed permanent transects (10x2m) at each site were surveyed for visual signs of disease and damage using the standardized method from the World Bank Coral Disease Working Group (58). MFPS was diagnosed as purple spots of approximately 1–3 mm (59). MFPS severity was estimated using the number of purple spots per colony, capped at 50 into a “50–100” category due to time constraints underwater. Tissue samples (1 cm x 2 cm) were collected from 1 healthy colony and 1 MFPS-diseased colony on each transect for use in disease diagnosis and amoebocyte analyses using histology (see below). We collected a single sample from the healthy colonies and two samples from each diseased colony, one with signs of MFPS and one asymptomatic for a total of N=9 samples and N=6 colonies per site (N=135 samples from N=90 colonies total, Permit # O-VS-PVS15-SJ-00671-24042014).

We included the following environmental and demographic variables: sea fan density, coral cover, water depth, temperature, colony size, and copper levels. Sea fan density could influence disease *via* transmission and was measured as the total number of sea fans on a 10 m x 2 m belt transect. Coral cover was measured on each transect as in Tracy et al. (44). We measured depth for each transect with depth gauges on dive gear. Comparable iButton and HOBO loggers collected temperature readings for each site every 2 h from 11 June to 28 November 2015 (60; **Table S4**). Colony size was the product of height and width, both measured with a PVC guide in the field to the nearest 1 cm. We used site level sediment copper concentrations from Pait et al. (61) since metal pollution can influence coral disease and immunity (13).

### Field and Laboratory Analyses

#### Amoebocyte Density and Diagnosis of Infections

Histology samples from both the field and laboratory were fixed in formalin, decalcified in a 50/50 mix of 10% citric acid and 30% formic acid, rinsed for 6 h, and embedded, sectioned (5 μm sections), and stained with hematoxylin and eosin by the Cornell University Veterinary Histology Laboratory. Signs of MFPS were used to collect samples from the field, but actual infection status (as opposed to external signs) was determined using light microscopy based on the presence of fungal hyphae and copepods within sea fan tissues as described by Ivanenko et al. (42) and Tracy et al. (44) (**Figure 1**). Copepods are closely associated with MFPS but histological diagnosis provides confirmation of infection, whereas fungal hyphae can occur in locations without external signs and rely on histological diagnosis. Based on the knowledge that multiple fungal species infect sea fans (40), we treat fungal hyphae visualized in histology as a general group for the purposes of this study. Copepod severity for the field and laboratory samples was determined from the number of copepods in the sea fan tissue.

We calculated amoebocyte density as a metric of cellular immunity using pictures of sea fan tissue taken at 40x with an Olympus BH-2 compound light microscope system. Photo locations were chosen using methods to control for researcher bias. For the laboratory experiment, pictures were taken along an arc 3 mm from the point of injection marked by the right angle of the triangular sample, as there was insufficient tissue along an arc at 1 mm. Photos were taken at all locations with sufficient tissue, with an average of 5.15 locations per sample and ranging from three to eight (total N=412 photos for 80 samples). For field samples, eight photos were taken at randomized locations in a numbered grid chosen using a random number generator for healthy samples (as in 49). For diseased samples (those that were infected with fungus, copepod or both in histology-based diagnoses), photos were taken within 1 mm of parasitic infections in the four cardinal directions (top, bottom, left, right) to control for bias by removing researcher choice of location (25). If both parasites were present in a sample, the infection of distinct tissues allowed for measuring within a 1mm radius for each parasite separately. Amoebocyte density was measured as a percentage of the total area of mesoglea using ImageJ, as in Couch et al. (49). In brief, the researcher outlined the entire area of mesoglea in each photo and used ImageJ to calculate the area of this irregular polygon, then converted the image to black and white and calculated amoebocyte density as the black area in the mesoglea as a percent of the total area (49).

#### Statistical Analyses

All statistical analyses were conducted in R version 3.1.2 (62) using linear models in lme4 (63). We first assessed homogeneity of variance and re-scaled numeric variables (rescale in the arm package, 64). Model selection was performed using drop1 to sequentially drop terms that did not improve the model and the best model was selected using Akaike Information Criteria (AIC) first, then the Likelihood Ratio Test (LRT) (if models were nested) or parsimony in the case of similar AIC values. Model fit was assessed using piecewiseSEM when possible (65). Variance inflation factors were used to check collinearity in all best models with more than one predictor using the rms package in R (66). Model averaging was performed using MuMin for models within 6 AIC units and 95% weight (67, 68). We plotted the fitted vs. residual values, checked for overdispersion, and confirmed normality of residuals where appropriate.

##### (a) Laboratory Data

We omitted one of the 10 colonies from laboratory analyses based on heavy, pre-existing infections in both MFPS and asymptomatic samples. For amoebocyte density and gene expression, we tested the effect of each treatment (*Aspergillus* inoculation, existing copepod infection, both or neither). To evaluate gene expression, we first confirmed there were no significant predictors of the reference gene, EF1, for the three candidate genes (**Appendix S1: Table S5**). This enabled us to test all models for each candidate immune gene. We tested models with all combinations of treatments, as well with additive and interactive effects of time. We tested a competing set of models that grouped the single *Aspergillus* infections, single copepod infections, and co-infections into one group. These models tested whether immunity was best predicted by the presence of any type or number of parasites (i.e. the general parasite term, “parasite”). We also tested whether amoebocyte density and candidate immune gene expression were predicted by the presence of 0, 1, or 2 parasites with a model grouping the single *Aspergillus* and single copepod infections together (i.e. “parasite_single”) (**Appendix S1: Table S6**).

Amoebocyte density was square-root transformed to meet the linear model’s assumptions of normality and analyzed using a linear mixed model (LMM) with sample nested within colony as random effects. Candidate gene expression and reference gene expression were also analyzed with LMMs. For each gene set, we first determined the best fit random effects structure including random slopes for all predictors and colony as a random effect (**Appendix S1: Table S7**). T5A, IκB, and MMP expression were square-root transformed to meet the LMM’s assumptions of normality. We assessed the six pairwise correlations between amoebocyte density and the three candidate genes using Spearman’s rho and Holm’s Bonferroni correction.

Copepod severity was analyzed with colony as a random effect and a negative binomial distribution due to overdispersion (glmer.nb in lme4, 63). We tested co-infection, time, and their interaction as predictors.

##### (b) Field Data

We analyzed whether co-occurrence of the copepod and fungal hyphae was greater or less than expected by chance using a Chi Square analysis and the histology diagnoses from the 90 colonies (6 colonies x 15 sites). Parasite diagnoses from diseased and asymptomatic samples from colonies with signs of MFPS were combined for a single, colony-level disease status to be most biologically relevant. We then tested the environmental and demographic predictors of the copepod and the fungus using generalized linear models (GLMs) with a binomial distribution and a logit link function. For fungal prevalence, additive model was tested that included copepod infection, site, mean temperature or temperature variability (standard deviation), coral cover, sea fan density, copper sediment concentration (61), depth, sea fan colony size, and reproductive status (i.e. the presence of ovaries, spermaries, or neither). We tested predictors of copepod prevalence based on the visual signs of disease (MFPS) using the larger dataset of all colonies surveyed at the 15 sites (N=1636). Our previous studies have established the link between copepod infection and MFPS (13, 44). For MFPS prevalence, we used generalized linear mixed models (GLMMs) with site as a random effect. We also analyzed copepod/MFPS severity, i.e. the number of purple spots on the entire sea fan colony, using censReg models due to the cap at counting 50 spots from time constraints while diving (69). For MFPS prevalence and severity, we tested the following predictors: mean temperature or temperature variability, coral cover, sea fan density, copper sediment concentration (61), depth and sea fan colony size.

Amoebocyte density was analyzed for the 90 colonies as a function of infection status (colony-level fungal and copepod infection) and environmental and demographic predictors: mean temperature or temperature variability (standard deviation), coral cover, sea fan density, copper sediment concentration (61), sea fan colony size, site, and reproductive status. We used drop1 for sequential model selection to test an additive model with all predictors except depth (which was tested in a subset of models without the deepest site), as well as a model including an interaction between copepod and fungal infection to test the effect of co-infection. We analyzed amoebocyte density using GLMs with a negative binomial distribution to account for overdispersion.

## Results

### Laboratory Experiment

**Figure 2:**
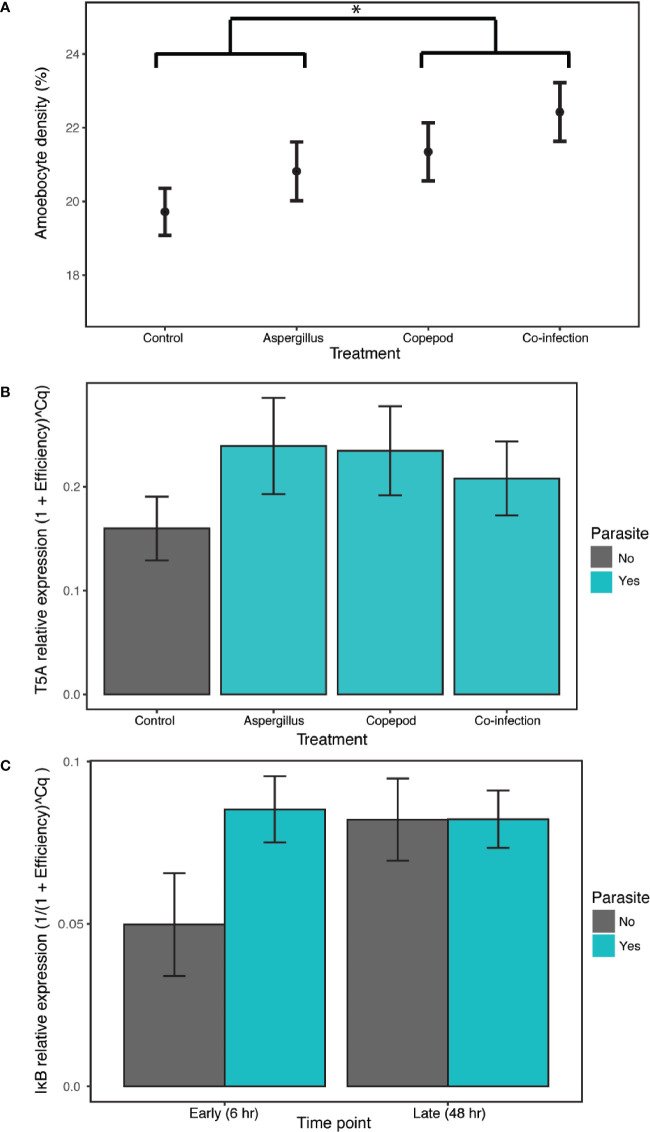
The clonally-replicated laboratory experiment was conducted using eight tissue samples from 10 colonies for each of the 8 treatments. The results of the experiment show **(A)** higher amoebocyte density during copepod infections (copepod and co-infection treatments) in comparison to the treatment with *Aspergillus* inoculation alone and the control. The 6- and 48-h time points are combined for each treatment as time was not a significant predictor. **(B)** Exposure to any type or number of parasites (i.e. the copepod, *Aspergillus* and co-infection treatments shown in green) induced T5A expression in the lab relative to the control. The 6- and 48-h time points are again combined as time was not a significant predictor. **(C)** IκB gene expression increased in the presence of any type or number of parasites in the early time point, though not significantly. IκB expression is combined across parasite treatments (copepod, *Aspergillus* and co-infection in green). Error bars are +/- 1SE. * denotes significance at alpha = 0.05.

There were elevated amoebocyte levels during copepod infections with an 8.16% increase relative to the control and single Aspergillus infections, which did not significantly induce a response. The best model of amoebocyte density in the lab experiment included only copepod presence, the only model that was significantly better than the null by the LRT and supported by model averaging (estimate= 0.0105, P= 0.049, CI 0.00242–0.208; **Appendix S1: Tables S8, S9**, **Figure S3**; **Figure 2A**).

T5A expression and, to a lesser extent, IκB expression indicate a host response to general parasite infection, though MMP does not. T5A expression increased with parasite infection of any kind, with the best model including the “parasite” term (i.e. combining the single parasite treatments and co-infections) and time as additive predictors and time as a random slope, though time was not significant (estimate= 0.0369, P= 4.4E-04, CI 0.0176–0.0562; **Appendix S1: Tables S10, S11**; **Figure S4**; **Figure 2B**). Results for IκB were mixed, as both “parasite_single” and “parasite” were supported as random slopes. The best model of IκB gene expression was the null in models with “parasite_single” as a random slope. However, there is weak support for time and infection as predictors of IκB expression, as the model with an interaction between the “parasite” term and time was the best by AIC in models with “parasite” as a random slope. The difference with and without parasites was marginally significant (**Appendix S1: Tables S12, S13**; parasite:time estimate= 0.0135, P= 0.0583; **Figure S5**; **Figure 2C**). For MMP, the best model was the null model (**Appendix S1: Table S14, Figure S6**). Amoebocyte density was positively correlated with T5A expression (rho= 0.411, P= 0.00228) and MMP expression (rho= 0.315, P= 0.0289) across all colonies and treatments, and T5A and MMP were also positively correlated with each other (rho= 0.394, P= 0.00335; **Appendix S1: Table S15**). Copepod severity was not predicted by time, co-infection, or their interaction (**Appendix S1: Table S16**).

### Field Survey

**Figure 3:**
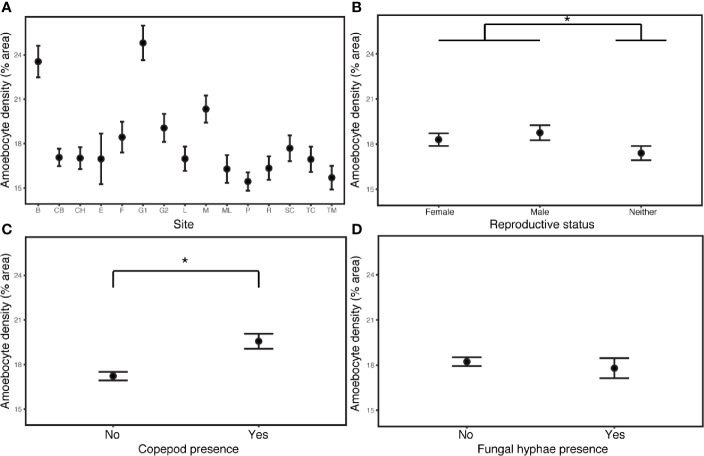
Amoebocyte density was measured in 135 samples from 90 wild colonies sampled across 15 sites in the field. **(A)** Site is the most influential predictor of amoebocyte density in field samples. **(B)** Reproductive sea fans have elevated amoebocyte density relative to sea fans lacking ovaries or spermaries. **(C)** Amoebocyte density increases with copepod infection, but **(D)** is unchanged in fungal infections. Error bars are +/- 1SE. * denotes significance at alpha = 0.05.

The field survey revealed that environment and demography joined parasitic infection as major drivers of disease and immunity. Constitutive amoebocyte density in the field samples (N=90) increased by 13.6% in copepod infections (estimate= 0.0870, P= 3.47E-10, CI 0.0597, 0.114), but was not predicted by the presence of fungal hyphae. The best model also included increasing amoebocyte density in reproductive sea fans (those with ovaries or spermaries) relative to sea fans lacking these structures (estimate= 0.0106, P= 1.84E-06, CI 0.0298, 0.0715), increasing amoebocyte density with coral cover (estimate= 0.178, P= 7.18E-05, CI 0.0891, 0.267), as well as differences across sites (**Appendix S1: Tables S17, S18**; **Figure 3**). Models without the deepest site did not support depth as a predictor of amoebocyte density (**Appendix S1: Table S19**).

**Figure 4:**
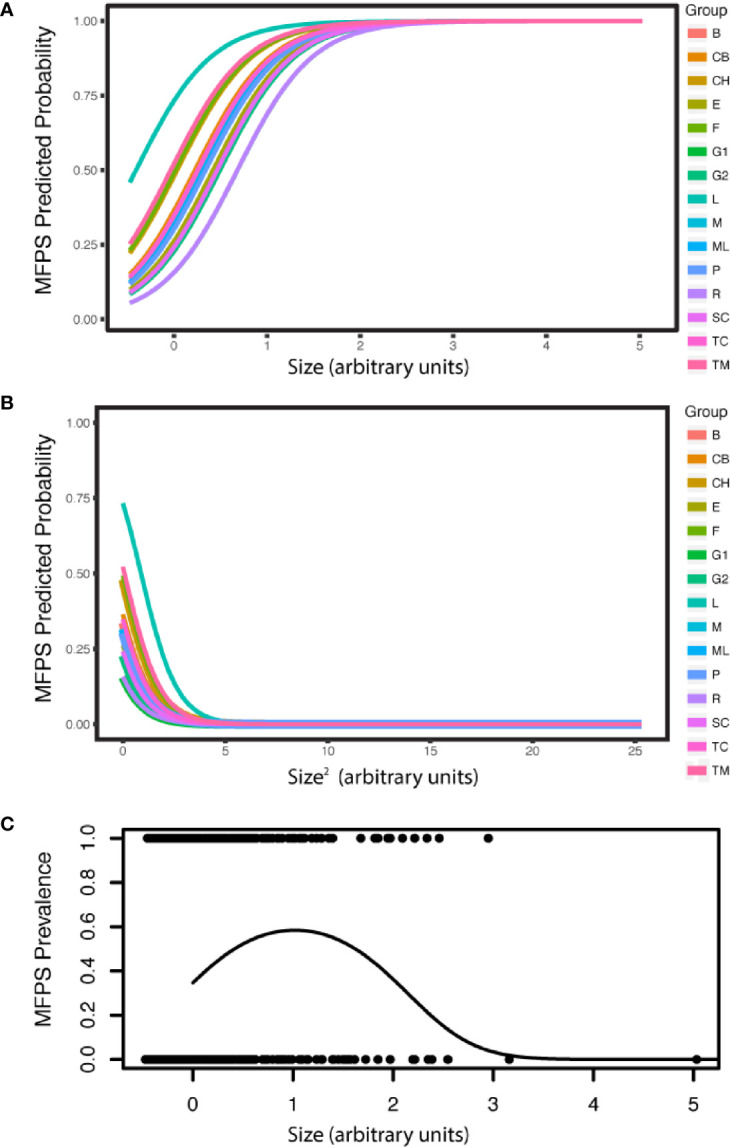
MFPS prevalence was evaluated in 135 samples from 90 wild colonies sampled across 15 sites in the field. MPFS prevalence **(A)** first increases with colony size, but **(B)** then decreases in the very largest colonies (predicted probabilities). The resulting overall pattern is a **(C)** a quadratic relationship [individual shown in **(A)** and **(B)** only]. The groups in the legend, i.e. the random effect in the GLMM, are the 15 reef sites where we sampled these wild colonies (**Appendix S1: Table S4**) and colony size is in arbitrary units because it is re-scaled in the arm package (58) for use in the GLMMs.

The prevalence of MFPS in the dataset of 1636 colonies was 31.1%, and was explained by sea fan colony size, temperature and coral cover. MFPS increased in larger fans (estimate= 2.47, P<2.00E-16, CI 2.06–2.88), decreased with temperature (estimate= -0.647, P= 0.0267, CI -1.22, -0.0747), and decreased non-significantly with coral cover (estimate= -0.396, P= 0.0911, CI -0.856, 0.0634). The presence of a quadratic term for colony size (size^2^) in the best model also indicates the relationship is a curve, with MFPS prevalence first increasing with colony size, then decreasing in the very largest sea fans (estimate= -1.14, P= 1.04E-13, CI -1.44, -0.84; **Appendix S1: Tables S20, S21**; **Figure 4**). MFPS severity was also best predicted by a quadratic size term (size: estimate= 0.834, P= 1.83E-08, CI 0.543–1.12; size^2^: estimate= -0.256, P= 0.0346, CI -0.493, -0.0185), in addition to site (**Appendix S1: Tables S22, S23**). Fungal hyphae were present in 13.4% of sea fan colonies based on histological analysis (N=90), but there were no significant predictors of fungal infection among the suite of environmental, demographic, and parasite predictors we analyzed, as the null model was better than the best additive model (Chi Sq. = 2.24, P= 0.134). Prevalence of co-infection was 8.89%, while 4.44% had only the fungus, 37.8% had only the copepod, and 48.9% had neither. The copepod parasite showed random co-occurrence with fungal hyphae (Chi squared= 1.39, P= 0.238, **Appendix S1: Figure S7**).

## Discussion

The host immune system mediates host-parasite interactions in natural populations amidst the influence of the environment and host demography. Previous eco-immunological studies have worked to uncover how immunity works in nature, demonstrating that factors as diverse as temperature, host age, reproductive status and co-infection with multiple parasites can all lead to variability in immunity among hosts (3, 4, 36). Our results from a paired laboratory experiment and field survey tackle this longstanding question and show that that immunity and disease are modulated by environment and demography in a wild population of sea fan octocorals. We first illustrate that sea fans exhibit altered expression of immune genes during infection with both parasites in the lab. The pre-existing nature of copepod infections could account for the increased amoebocyte densities relative to *Aspergillus* treatments, as chronic infections can lead to immune suppression or induction (70, 71). However, the field survey provides additional support for an asymmetric response because amoebocyte density was elevated in existing copepod infections and not in existing fungal infections. Host immunity in the field was explained in part by these parasitic infections. However, environmental and demographic factors, including sea fan reproductive status, coral cover and the reef site, also played an important role. The parasites’ random co-occurrence shows that immune induction by one parasite need not lead to negative co-occurrence in nature, which may be due to environmental and demographic drivers. Features of the wild sea fan population, colony size in particular, also influenced disease patterns by influencing MFPS prevalence and severity.

The laboratory experiment revealed that single infections and co-infections with the existing copepod infection and live *Aspergillus* inoculation affect candidate immune gene expression and amoebocyte density. Expression of candidate immune genes changes during infection with both parasites in the lab, with an increase of 38.9% in a putative pathogen recognition gene (T5A) in response to the combination of all *Aspergillus* and copepod treatments. There was also weak evidence of this pattern for IκB. Amoebocyte density was elevated by 8.16% during copepod infections across both time points (6 and 48 h), but did not respond significantly to *Aspergillus* inoculations at either time point. This difference in cellular immunity indicates that immune metrics depend on which parasite is present, with amoebocyte density activated during existing copepod infections and no change during fungal inoculation. While it is possible that the lack of an amoebocyte response to fungal infections could result from inoculations not leading to infections, the field results confirm the lack of amoebocyte response in fungal infections. Moreover, there are changes in gene expression with fungal inoculations in the laboratory experiment that suggest immune challenge alone elicits a response even if an infection does not establish. The distinct cellular immune response for the copepod and fungus thus persists. Parasite-specific immune responses exist in many other systems, including buffalo, rabbits and damselflies (30, 72, 73). The degree to which hosts tailor their responses to different pathogens is important for assessing immune trade-offs for hosts fighting multiple parasites. For example, macroparasite infections in mice increase macroparasite-specific responses at the expense of defenses that protect hosts against microparasites, thereby benefitting microparasites as they attack hosts (74). There is similar evidence in invertebrates, with trade-offs observed between bactericidal and phagocytic branches of the effector response (75). The results also show that sea fan immunity differs in single vs. co-infections, but only for the *Aspergillus*: the amoebocyte response was significantly induced when *Aspergillus* was inoculated in sea fans with the existing copepod infection, but not in single infections. In addition to characterizing immunity during parasitic infection, we also detected a positive correlation between amoebocyte density, T5A and MMP expression irrespective of treatment. The shared pattern for these three metrics across all colonies and treatments is helpful because multiple immune metrics are more reliable than single metrics in assessing a host’s immune status or response (4, 76). Given that parasitic infection is not a requirement, it is possible that these three responses are activated by a diversity of stressors, whether separately or together, and are generally inflammatory in response to damage, infection and other stressors.

The responses of the candidate immune genes further our understanding of coral immune pathways. We confirm the induction of T5A to two other known sea fan parasites beyond the labyrinthulid (14, 17). We cannot yet conclude that T5A is functioning as a lectin involved in pathogen recognition in *G. ventalina*. Regardless, this gene appears to have a non-specific response since it was induced by all parasites. If it is indeed involved in pathogen recognition in *G. ventalina*, it is possible that differential responses by sea fans to these parasites may only appear after this recognition stage. Alternatively, distinguishing between parasites may be accomplished by multiple lectins and not just T5A, several of which occur in cnidarians (14). Our experimental design cannot determine the role of injection damage in gene expression, but it is possible that T5A expression increases not only with parasite exposure relative to control injection with SSW, as indicated here, but also with any injection relative to non-injected samples. Such general responsiveness to stress, including damage and infection, is common in invertebrates (26).

Similar to the pattern of T5A activation, IκB showed increasing expression, though not significantly, in the presence of all the parasite treatments (single and co-infections). Although the function of IκB in *G. ventalina* is unknown, elevated IκB expression during infection could indicate changes in the Toll pathway (or a Toll-like pathway) as part of the sea fan response to the macroparasites in this study. For example, increased expression of this NF-κB inhibitor could decrease host investment in the Toll pathway, which generally targets microparasites, in favor of a cellular response (i.e. amoebocytes) targeting macroparasites like those in this study (51). Such trade-offs between microparasite and macroparasite defenses are well-known in vertebrates, with some evidence in invertebrates (74, 75, 77).

For MMP, the lack of immune activation during infection with the copepod and *Aspergillus* parasites contrasts with the differential expression detected in response to the labyrinthulid (17). This may mean that MMP expression is parasite-specific, but the time point of expression could also matter. We chose the 48-h time point instead of the 24-h time point in Burge et al. (17) because amoebocyte density peaks at 48 h in a previous laboratory study of *G. ventalina* using exposure to grafts of sea fans with *Aspergillus*-like lesions (49). This alternate time point may account for the discrepancy in MMP gene expression between the studies. Gene expression and amoebocyte density are temporally dynamic and, while an improvement, assessing two time points is still unlikely to fully capture the response in these metrics. High variability between sea fan colonies, observed here and in previous studies (**Appendix S1: Figure S8**; 9, 49), could also account for the lack of an overall response by MMP. The lack of a response from MMP could mean that wound healing or the other immune-related functions of this gene in sea fans are not at play for the copepod and the *Aspergillus* at the selected time points, or that this pathway is activated through different genes for these parasites.

Translating the laboratory findings to a field context, our objective was to determine whether immunity and disease in the field depend on environmental and host demographic factors and to characterize parasite co-occurrence. Disease metrics in the field were indeed influenced by features of a natural population, as sea fan colony size predicted disease prevalence and severity for MFPS. The dominant role of sea fan colony size in predicting MFPS prevalence and severity illustrates that demography is critical for disease patterns. It also aligns with our previous work showing colony size predicts sea fan copepod infections (13, 44), and widespread findings of increases in disease prevalence with host age or size in corals, seagrass, and mammals (29, 44, 78, 79). Age and size effects are of interest in eco-immunology because they influence immune function in nature and can be used to study the influence of broader ecological phenomena, including life history, resource allocation and senescence (3, 29). In the present study, the increase in disease with host colony size is not due to immune senescence with age because size, a proxy for age in sea fans, is not a predictor of sea fan immunity in the field. Larger sea fans could have greater exposure from increased ingestion (if exposure is through feeding), a longer time to accumulate parasites, and/or greater targeting of a larger resource by parasites. The decline in MFPS prevalence in the largest sea fans could indicate mortality of sea fans that were infected heavily or for a long time.

Amoebocyte density in the field samples was best predicted by a combination of infection status, reproductive status, site and coral cover. Copepod infection and reproductive status had similar effects, whereas the influence of coral cover and site-specific factors on immunity was more pronounced. Site differences in immunity could result from host genetic differences across a metapopulation, which is compelling given high variability between sea fan colonies within a site and genetic differentiation across short distances (49, 80). Differences in immunity across sites has previously been identified, for example, in wild sticklebacks (81). A recent study of mice also found that demographic factors can supersede the effects of parasitism on immune function in the field. Interestingly, site variation in mouse immunity was not attributable to genetic factors, but rather body condition and age, suggesting that different food resources at different sites could affect immune variation in the wild (29). The differences in sea fan immunity across the 15 reef sites in the present study could be genetic or result from additional environmental factors not measured herein. Coral cover is one characteristic of reef sites that is linked to increased amoebocyte density herein. This finding aligns with terrestrial examples in which poor habitat quality affects immunity (4), and may mean that defenses are impaired on poorer quality reefs. The effect of coral cover may also be serving as a general indicator, as improved site quality in terms of water quality, food and light could lead to both increased coral cover and an increase in constitutive immunity in healthier colonies.

Reproductive status, another driver of sea fan amoebocyte density, also influences immunity in a variety of organisms, from fish to mice and rabbits (3, 29, 81). One of the tenets of ecological immunology is that physiological constraints prevent organisms from maximizing reproduction and defenses against all parasites, which can have far-reaching consequences for survival and evolution (3, 82). This pattern appears both in females, e.g. with reduced immunity during reproduction, and in males, e.g. with lower immune activity in mated males (72, 82, 83). There is also evidence for such trade-offs in corals, as hermaphrodites have lower constitutive immunity, and corals with fast-paced life histories appear to invest in less costly immune pathways (84). Despite the prevalence of this pattern, some studies fail to detect a negative relationship between reproductive status and immunity (83, 85). Our data are unusual in that we detect a positive relationship between reproductive status and immunity in sea fans. This is the opposite result from the expected trade-off between these two costly physiological processes (3). Explanations could include sufficient resources for both processes or an unmeasured cost along another axis, such as a different immune metric or growth (4, 76). Overall, the importance of these various site-specific and demographic factors highlights specific routes through which the environment and demography contribute to host immunity and parasite prevalence.

In light of the multiple drivers of host immunity, it is possible that reproductive status, coral cover and site differences overwhelm the effect of immune-mediated interactions between parasites on co-occurrence patterns in the field. This could account for the parasites’ random co-occurrence. Even if the immune activation we observed leads to immune-mediated antagonism between the two parasites, this may not manifest as negative co-occurrence because of the greater influence of ecological factors. An alternate hypothesis for the disconnect between immune profiles and parasite co-occurrence is that, despite their distinct resource use within a sea fan colony, the parasites both target high quality or highly susceptible hosts. In this case, the negative pattern expected from immune-mediated antagonism combined with the positive force of common resource use could equilibrate as random co-occurrence. There are several systems where competing forces have created such unexpected co-occurrence patterns (27, 30, 33, 34). Another possibility is that host mortality drives patterns of co-occurrence (30, 32). Whole-colony mortality is infrequent over the scale of a few years in these sea fans (Tracy et al., unpublished data), but colony mortality may not be the correct metric to evaluate the impact of mortality in sea fans. This is a strong example of the curiosities of studying co-infection in colonial organisms because sea fan mortality could be measured as whole-colony mortality or as polyp mortality, but the latter is rarely measured and is difficult to track. Yet another possibility is that the colonial architecture of colonies leads to strong separation between polyps, such that infections in separate polyps do not interact at all through resource or immune pathways, in contrast to traditional co-infection theory (28). Further research is needed to more clearly shape expectations for parasite interactions during co-infection in colonial organisms.

We evaluated the consequences of co-infection on infection severity in the laboratory to shed light on the nature of the parasite interaction, as measured by the impact of co-infection on parasite fitness in terms of parasite counts. The laboratory experiment provided an opportunity to assess the impact of *Aspergillus* treatment on the severity of copepod infection with a clear order of infection that is helpful for studying directional effects. We found that *Aspergillus* treatment had no effect on the number of copepods in infected tissue in the laboratory. It is unlikely that the 48-h time period was long enough to observe effects given the long-standing nature of copepod infections (13). Future approaches could include longer exposure or pursue isolating the copepod to conduct experiments on how the order of parasite infection, i.e. priority, influences parasite interactions. Investigating priority effects in particular is a key ingredient for understanding parasite coexistence (86).

This study is the first investigation of octocoral or Scleractinian coral immunity in a multi-parasite system. By uniting field surveys with laboratory experimentation and multiple immune metrics, we begin to uncover how sea fan immunity operates in a natural population. Site-specific factors and sea fan demography emerged as two key players in sea fan immunity and disease amidst co-infection. This exploration of sea fan immunity in a multi-parasite system advances the understanding of how immunity functions in natural populations, raises questions about co-infection theory in colonial organisms, and provides urgently needed information on the conditions that influence disease in an ecologically important host species.

## Data Availability Statement

The datasets presented in this study can be found in online repositories. The names of the repository/repositories and accession number(s) can be found below: https://datadryad.org/stash (https://doi.org/10.5061/dryad.q573n5tg0) (87).

## Author Contributions

AT designed the study. AT and EW conducted field surveys and experiments, and AT and CB conducted laboratory analyses. All authors contributed to the article and approved the submitted version.

## Funding

AT was supported by the National Science Foundation Graduate Research Fellowship Program (DGE-1650441); Sigma Xi, Cornell Chapter; a National Geographic Young Explorers Young Explorers Grant; a Cornell Graduate School Research Travel Grant; the Betty Miller Francis ‘47 Fund for field research; and the Cornell Department of Ecology and Evolutionary Biology Paul P. Feeny Fund. EW was partially funded by NSF (IOS#1017510) and the Department of Marine Sciences, University of Puerto Rico at Mayagüez. Logistical Support, lab and office space, was provided by EW and the Department of Marine Sciences, University of Puerto Rico at Mayagüez. Support was provided by start-up funds to CB to the Institute of Marine and Environmental Technology from the University of Maryland Baltimore County and the University of Maryland Baltimore.

## Conflict of Interest

The authors declare that the research was conducted in the absence of any commercial or financial relationships that could be construed as a potential conflict of interest.
