# Supplementary material for: Ecological Factors Mediate Immunity and Parasitic Co-Infection in Sea Fan Octocorals
Source: Front Immunol. 2021 Jan 11;11:608066. doi: 10.3389/fimmu.2020.608066 (PMC7829190; doi:10.3389/fimmu.2020.608066)
Supplement: Supplementary file 1 [file DataSheet_1.docx]

**Immunity and ecological factors mediate parasitic co-infection in sea fan octocorals**

Allison M. Tracy^1*^, Ernesto Weil^2^, Colleen A. Burge^3^

*Frontiers in Immunology*

**Appendix S1**

**Fig. S1:** Experimental design: (A) Eight samples were collected for each of 10 colonies in the field (4 healthy and 4 copepod-infected samples) for use in the laboratory experiment. (B) For each colony, two healthy samples and two copepod-infected samples were inoculated with *Aspergillus,* creating 4 treatments: control, copepod-infected (denoted by circle), *Aspergillus-*inoculated (denoted by asterisk), and copepod-infected + *Aspergillus*-inoculated (with both circle and asterisk). Eight samples were used for each colony to have two time points (6 hours and 48 hours) for each colony in each treatment.


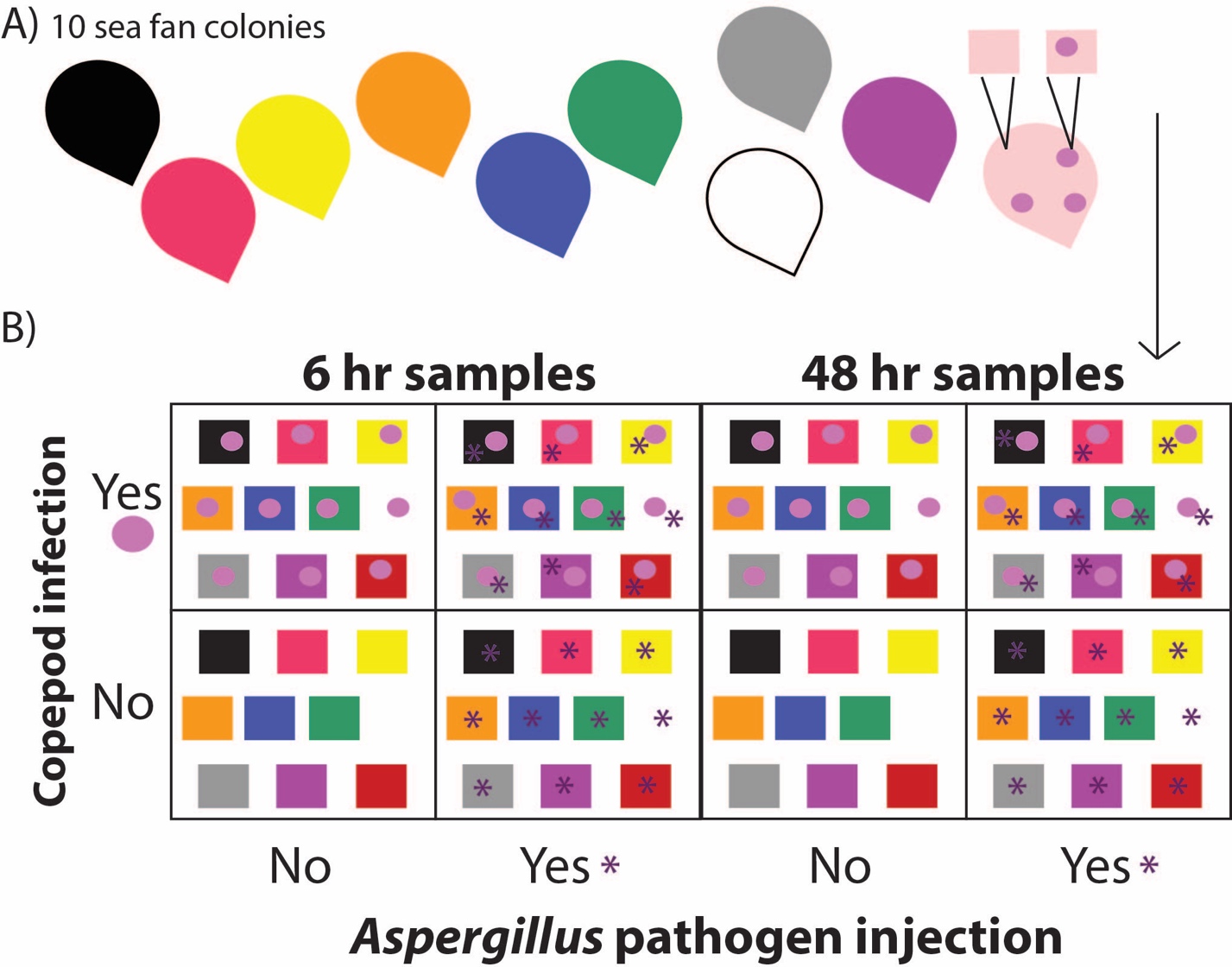


**Table S1:** Candidate immune genes identities and primers. (GenBank accession numbers pending).

| **Name** | **Swiss Prot ID** | ***G. ventalina* transcriptome ID** | **Direction** | **Primer sequence** | **Length** |
| --- | --- | --- | --- | --- | --- |
| **EF1**  *Elongation Factor 1* | A5D989 | 75614 | Forward | ACCGCTGCTAGTTTGGATGT | 20 |
|  |  |  | Reverse | TGATGAAACCGACATGGCTA | 20 |
| **T5A**  *Tachylectin 5A* | Q9U8W8 | 42227 | Forward | ATTTCGGTTTTCCTCAACGA | 20 |
|  |  |  | Reverse | CCTGGTGGTATGCTGCTTG | 19 |
| **MMP**  *Matrix metalloproteinase* | Q5RES1 | 81521 | Forward | ACCGTATTTCTGGACGTTGG | 20 |
|  |  |  | Reverse | CGGTTTTTCCATCTCGTCTT | 20 |
| **IκB**  *NF-κB Inhibitor cactus* | In sub-mission to GenBank | N/A | Forward | TCGAAGCATTGAGGTGTTGGA | 21 |
|  |  |  | Reverse | GACGTGCGATATTTGCTGGC | 20 |

**Table S2:** Nanodrop quality and quantity data for all samples

| **Sample** | **Timepoint of nanodrop** | **Concentration (ng/uL)** | **260/280** | **260/230** |
| --- | --- | --- | --- | --- |
| **A10L** | pre DNAse | 1696.5 | 2.04 | 2.55 |
|  | post DNAse | 321.1 | 2.03 | 2.43 |
| **A9L** | pre DNAse | 1465.1 | 2.08 | 2.25 |
|  | post DNAse | 302.3 | 2.27 | 2.11 |
| **N6L** | pre DNAse | 2148.1 | 2.07 | 2.44 |
|  | post DNAse | 364.4 | 2.05 | 2.26 |
| **M6E** | pre DNAse | 3655.1 | 2.12 | 2.36 |
|  | post DNAse | 341.1 | 2.03 | 2.52 |
| **M9E** | pre DNAse | 1744 | 2.13 | 2.55 |
|  | post DNAse | 494.4 | 2.07 | 2.49 |
| **N2L** | pre DNAse | 2623.3 | 2.11 | 2.37 |
|  | post DNAse | 369.3 | 2.05 | 2.4 |
| **M2E** | pre DNAse | 2092.7 | 2.07 | 2.53 |
|  | post DNAse | 370.4 | 2.08 | 2.13 |
| **A1E** | pre DNAse | 2312.1 | 2.11 | 2.52 |
|  | post DNAse | 379.2 | 2.22 | 2.13 |
| **MA6E** | pre DNAse | 1520.7 | 2.07 | 1.88 |
|  | post DNAse | 395.5 | 2.12 | 1.6 |
| **MA1E** | pre DNAse | 2668 | 2.08 | 2.45 |
|  | post DNAse | 343.3 | 2.16 | 2.2 |
| **M2L** | pre DNAse | 1112.8 | 2.04 | 2.49 |
|  | post DNAse | 395.6 | 2.23 | 2.13 |
| **N10L** | pre DNAse | 2489.7 | 2.1 | 2.36 |
|  | post DNAse | 367.8 | 2.19 | 2 |
| **A9E** | pre DNAse | 1220 | N/A | N/A |
|  | post DNAse | 932.9 | 2.03 | 1.89 |
| **N9L** | pre DNAse | 1280 | N/A | N/A |
|  | post DNAse | 1103.6 | 2.03 | 2.25 |
| **A2L** | pre DNAse | 1140 | N/A | N/A |
|  | post DNAse | 973.2 | 2.02 | 2.22 |
| **A1L** | pre DNAse | 560 | N/A | N/A |
|  | post DNAse | 564.9 | 2.14 | 1.94 |
| **N10E** | pre DNAse | 680 | N/A | N/A |
|  | post DNAse | 728.9 | 1.97 | 2.14 |
| **MA2E** | pre DNAse | 886 | N/A | N/A |
|  | post DNAse | 737.1 | 1.98 | 2.06 |
| **MA9L** | pre DNAse | 3209.3 | 2.09 | 2.36 |
|  | post DNAse | 356.5 |  |  |
| **MA10L** | pre DNAse | 3145.2 | 2.08 | 2.28 |
|  | post DNAse | 337 |  |  |
| **MA6L** | pre DNAse | 2308.3 | 2.12 | 1.74 |
|  | post DNAse | 374.1 |  |  |
| **M6L** | pre DNAse | 3306.4 | 2.07 | 2.43 |
|  | post DNAse | 335 |  |  |
| **M9L** | pre DNAse | 1726.6 | 2.08 | 2.33 |
|  | post DNAse | 346.3 |  |  |
| **M10L** | pre DNAse | 1783.8 | 2.09 | 2.49 |
|  | post DNAse | 396.9 |  |  |
| **N9E** | pre DNAse | 1067 | 2.06 | 2.51 |
|  | post DNAse | 405 | 2.01 | 2.26 |
| **N6E** | pre DNAse | 1926.4 | 2.1 | 2.46 |
|  | post DNAse | 360.9 | 2.05 | 2.24 |
| **M1L** | pre DNAse | 2370.4 | 2.13 | 2.32 |
|  | post DNAse | 343 | 1.99 | 2.19 |
| **MA1L** | pre DNAse | 3112.8 | 2.08 | 2.2 |
|  | post DNAse | 403.5 | 2.01 | 2.03 |
| **A6E** | pre DNAse | 2584.7 | 2.09 | 2.29 |
|  | post DNAse | 353.4 | 2.15 | 1.99 |
| **A10E** | pre DNAse | 2212.9 | 2.08 | 2.34 |
|  | post DNAse | 370.1 | 2.08 | 1.93 |
| **N2E** | pre DNAse | 3810.7 | 2.07 | 2.36 |
|  | post DNAse | 314.2 | 2.06 | 2.13 |
| **MA10E** | pre DNAse | 2740.9 | 2.09 | 2.45 |
|  | post DNAse | 327 | 2.11 | 2.09 |
| **A2E** | pre DNAse | 2722.8 | 2.07 | 2.39 |
|  | post DNAse | 366 | 1.96 | 2.06 |
| **MA9E** | pre DNAse | 3185.4 | 2.08 | 2.34 |
|  | post DNAse | 348.9 | 2.12 | 2.09 |
| **N1E** | pre DNAse | 2694.7 | 2.09 | 2.39 |
|  | post DNAse | 393.3 | 2.14 | 2.1 |
| **M1E** | pre DNAse | 3441.5 | 2.09 | 2.33 |
|  | post DNAse | 408 | 2.17 | 2.19 |
| **A6L** | pre DNAse | 3680.7 | 2.08 | 2.28 |
|  | post DNAse | 358.5 | 2.08 | 1.99 |
| **N1L** | pre DNAse | 2493.7 | 2.17 | 2.37 |
|  | post DNAse | 357.6 | 2.15 | 1.99 |
| **M10E** | pre DNAse | 3686.8 | 2.1 | 2.27 |
|  | post DNAse | 511.7 | 2.12 | 1.96 |
| **MA2L** | pre DNAse | 3758.4 | 2.08 | 2.42 |
|  | post DNAse | 415.2 | 2.02 | 1.98 |
| **MA4E** | pre DNAse | 3291.4 | 2.08 | 2.4 |
|  | post DNAse | 375.9 | 2.1 | 2.14 |
| **A3L** | pre DNAse | 3631.5 | 2.09 | 2.34 |
|  | post DNAse | 359.8 | 2.18 | 2.13 |
| **M7L** | pre DNAse | 2382.7 | 2.11 | 2.49 |
|  | post DNAse | 350.1 | 2.13 | 2.14 |
| **M8L** | pre DNAse | 1765.9 | 2.1 | 2.56 |
|  | post DNAse | 371.7 | 2.11 | 2.34 |
| **M5E** | pre DNAse | 2208.9 | 2.08 | 2.51 |
|  | post DNAse | 410.5 | 2.14 | 2.21 |
| **A4E** | pre DNAse | 2834.3 | 2.11 | 2.47 |
|  | post DNAse | 393.2 | 2.09 | 2.16 |
| **N8L** | pre DNAse | 2186 | 2.13 | 2.45 |
|  | post DNAse | 385.3 | 2.11 | 2.14 |
| **A5L** | pre DNAse | 2676.5 | 2.09 | 2.44 |
|  | post DNAse | 401.9 | 2.02 | 2.16 |
| **MA8L** | pre DNAse | 2013.1 | 2.13 | 2.41 |
|  | post DNAse | 378 | 2.01 | 2.03 |
| **MA8E** | pre DNAse | 1617.7 | 2.17 | 2.41 |
|  | post DNAse | 379.5 | 1.97 | 2.07 |
| **M4E** | pre DNAse | 1828.5 | 2.15 | 2.46 |
|  | post DNAse | 374.8 | 2.05 | 2.28 |
| **N4E** | pre DNAse | 2830.8 | 2.13 | 2.22 |
|  | post DNAse | 371.4 | 2.03 | 1.94 |
| **A8L** | pre DNAse | 2314.7 | 2.15 | 2.46 |
|  | post DNAse | 340.5 | 2.13 | 2.24 |
| **MA5L** | pre DNAse | 2239.8 | 2.15 | 2.35 |
|  | post DNAse | 405 | 2.11 | 2.14 |
| **MA7L** | pre DNAse | 2102.1 | 2.17 | 2.45 |
|  | post DNAse | 335.6 | 2.24 | 2.29 |
| **A8E** | pre DNAse | 2015.1 | 2.17 | 2.45 |
|  | post DNAse | 404 | 2.01 | 2.29 |
| **M3E** | pre DNAse | 2203.1 | 2.16 | 2.38 |
|  | post DNAse | 365.8 | 2.11 | 2.15 |
| **MA5E** | pre DNAse | 2777.2 | 2.16 | 2.47 |
|  | post DNAse | 374.5 | 2.04 | 2.14 |
| **M3L** | pre DNAse | 2988.1 | 2.09 | 2.45 |
|  | post DNAse | 308.9 | 2.07 | 2.02 |
| **M5L** | pre DNAse | 2751.2 | 2.09 | 2.37 |
|  | post DNAse | 382.6 | 2.14 | 2 |
| **A7L** | pre DNAse | 3005.6 | 2.11 | 2.39 |
|  | post DNAse | 337.3 | 1.95 | 2.04 |
| **A7E** | pre DNAse | 2740 | 2.1 | 2.49 |
|  | post DNAse | 323.3 | 2.07 | 2.14 |
| **M8E** | pre DNAse | 1843.8 | 2.1 | 2.46 |
|  | post DNAse | 357.6 | 2 | 2.13 |
| **MA3E** | pre DNAse | 2161.3 | 2.12 | 2.47 |
|  | post DNAse | 306.5 | 2.13 | 2.09 |
| **N3L** | pre DNAse | 2493.6 | 2.12 | 2.46 |
|  | post DNAse | 335 | 2.12 | 2.08 |
| **MA4L** | pre DNAse | 1913.8 | 2.11 | 2.5 |
|  | post DNAse | 328.8 | 2.15 | 2.09 |
| **N7L** | pre DNAse | 2152.8 | 2.08 | 2.4 |
|  | post DNAse | 363.4 | 2.05 | 2.2 |
| **N3E** | pre DNAse | 2404.1 | 2.11 | 2.35 |
|  | post DNAse | 362.3 | 2.13 | 2.06 |
| **MA7E** | pre DNAse | 2689.3 | 2.1 | 2.24 |
|  | post DNAse | 373.4 | 2.02 | 2.04 |
| **M7E** | pre DNAse | 2240.1 | 2.11 | 2.25 |
|  | post DNAse | 337.1 | 2.09 | 1.91 |
| **N8E** | pre DNAse | 1095.4 | 2.14 | 2.44 |
|  | post DNAse | 356.3 | 2.05 | 2.24 |
| **N7E** | pre DNAse | 1812.6 | 2.1 | 2.43 |
|  | post DNAse | 364.8 | 2.17 | 2.26 |
| **A5E** | pre DNAse | 3025.4 | 2.08 | 2.43 |
|  | post DNAse | 396 | 2.1 | 2.3 |
| **M4L** | pre DNAse | 2391.9 | 2.09 | 2.41 |
|  | post DNAse | 321.8 | 2.08 | 2.11 |
| **A4L** | pre DNAse | 2814.4 | 2.1 | 2.33 |
|  | post DNAse | 362.2 | 2.03 | 2.25 |
| **MA3L** | pre DNAse | 2638.1 | 2.09 | 2.27 |
|  | post DNAse | 388.6 | 2.12 | 2.21 |
| **N4L** | pre DNAse | 3368.6 | 2.06 | 2.26 |
|  | post DNAse | 398.3 | 2.03 | 1.95 |
| **N5L** | pre DNAse | 1846.9 | 2.06 | 2.04 |
|  | post DNAse | 407.7 | 2.1 | 1.82 |
| **N5E** | pre DNAse | 1196.6 | 2.03 | 2.39 |
|  | post DNAse | 392.9 | 2.07 | 2.15 |
| **A3E** | pre DNAse | 1547.5 | 2.06 | 2.35 |
|  | post DNAse | 368.2 | 2.12 | 1.86 |

**Table S3:** Efficiencies of genes from PCR Miner, averaged across Miner runs

| **Gene** | **Efficiency** |
| --- | --- |
| EF1 | 0.882 |
| T5A | 0.803 |
| IκB | 0.860 |
| MMP | 0.853 |

**Fig. S2:** Map of survey sites in The Natural Reserve of La Parguera and in Guánica, on the southwest coast of Puerto Rico.

**Table S4:** GPS coordinates of sites in La Parguera and Guánica, Puerto Rico with the abbreviation, temperature logger type and copper concentration (Pait et al. 2007).

| **Reef site name and #** | **Latitude** | **Longitude** | **Temperature Logger** | **Copper sediment concentration range** |
| --- | --- | --- | --- | --- |
| **Turrumotico (#1) “TC”** | 17° 55.743' | 66° 58.487'' | Use Turrumote | 0.0-0.9 μg/g |
| **Turrumote (#2) “TM”** | 17° 56.097' | 67° 01.130'' | HOBO | 1.0-1.9 μg/g (Indicator Kriging, Pait et al. 2007) |
| **Laurel Patch (#3) “L”** | 17° 56.537' | 67° 04.057' | Use Media Luna | 0.0-0.9 μg/g |
| **Media Luna (#4) “ML”** | 17° 56.096' | 67° 02.911' | HOBO | 2.0-4.1 μg/g |
| **Pelotas**  **(#5) “P”** | 17° 57.446'' | 67° 04.183' | HOBO | 2.0-4.1 μg/g |
| **Caballo Blanco**  **(#6) “CB”** | 17° 57.831' | 67° 02.940' | iButton | 2.0-4.1 μg/g |
| **Enrique**  **(#7) “E”** | 17° 57.294' | 67° 02.608' | HOBO | 4.2-80.6 μg/g |
| **Corral Channel**  **(#8) “CH”** | 17° 56.959' | 66° 59.938'' | iButton | 4.2-80.6 μg/g |
| **Fosfo Bay**  **(#9) “F”** | 17° 57.544' | 67° 00.826' | Use Channel | 4.2-80.6 μg/g |
| **Mario reef**  **(#10) “M”** | 17° 57.170' | 67° 03 387' | HOBO | 4.2-80.6 μg/g |
| **Buoy**  **(#11) “B”** | 17° 53.291'' | 66° 59.884' | HOBO | 0.0-0.9 μg/g (Indicator Kriging, Pait et al. 2007) |
| **Romero**  **(#12) “R”** | 17° 56.830' | 66° 59.431' | iButton | 4.2-80.6 μg/g (Indicator Kriging, Pait et al. 2007) |
| **San Cristobal**  **(#13) “SC”** | 17° 56.497' | 67° 04.497' | Use Pelotas | 1.0-1.9 μg/g (Indicator Kriging, Pait et al. 2007) |
| **Guánica 1**  **(#14) “G1”** | 17° 56.862' | 66° 54.087' | iButton | 1.0-1.9 μg/g (Indicator Kriging, Pait et al. 2007) |
| **Guánica 2**  **(#15) “G2”** | 17° 56.529' | 66° 54.855' | Use Guánica 1 | 1.0-1.9 μg/g |

**Table S5**: None of the infection terms, time or interactions predicted EF1 expression for any of the subsets (NS = not significant; DF = degrees of freedom; LRT = likelihood ratio test). Models include colony as a random intercept. No random slopes were supported by model comparison.

| **EF1 (T5A samples)** | | | **EF1 (IκB samples)** | | | **EF1 (MMP samples)** | | |
| --- | --- | --- | --- | --- | --- | --- | --- | --- |
| **Model** | **DF** | **Delta AIC** | **Model** | **DF** | **Delta AIC** | **Model** | **DF** | **Delta AIC** |
|  |  |  |  |  |  |  |  |  |
| Parasite*time [NS predictors] | 6 | 0 | Parasite*time [NS predictors] | 6 | 0 | Aspergillus*time [not better than null by LRT] | 6 | 0 |
| Null | 3 | 2.01 | Parasite_single*time [NS predictors] | 8 | 0.79 | Aspergillus*time+copepod [not better than null by LRT] | 7 | 1.5 |
| Aspergillus*time | 6 | 2.4 | Aspergillus*time [NS predictors] | 6 | 3.05 | Null | 3 | 1.7 |
| Copepod | 4 | 2.4 | Copepod*Asperigllus*time [NS predictors] | 10 | 4.61 | Parasite*time | 6 | 2.33 |
| Parasite | 4 | 2.6 | Aspergillus*time+copepod [NS predictors] | 7 | 5.01 | Parasite | 4 | 3.07 |
| Aspergillus*time+copepod | 7 | 2.63 | Copepod*time [NS predictors] | 6 | 5.59 | Copepod | 4 | 3.26 |
| Parasite_single*time | 8 | 3.58 | Parasite+time [NS predictors] | 5 | 6.25 | Aspergillus | 4 | 3.55 |
| Aspergillus | 4 | 4.01 | Copepod+time [NS predictors] | 5 | 7.2 | Parasite+time | 5 | 4.09 |
| Copepod+time | 5 | 4.06 | Aspergillus+time [NS predictors] | 5 | 7.21 | Copepod+time | 5 | 4.28 |
| Parasite+time | 5 | 4.26 | Parasite_single+time [NS predictors] | 6 | 7.38 | Aspergillus+time | 5 | 4.57 |
| Copepod+Aspergillus | 5 | 4.39 | Copepod*time+Aspergillus [NS predictors] | 7 | 7.55 | Parasite_single | 5 | 5.04 |
| Parasite_single | 5 | 4.55 | Null | 3 | 8.07 | Copepod+Aspergillus | 5 | 5.1 |
| Copepod*time | 6 | 5.03 | Parasite | 4 | 9.15 | Parasite_single*time | 8 | 5.43 |
| Copepod*Aspergillus | 6 | 5.61 | Copepod+Aspergillus+time | 6 | 9.17 | Parasite_single+time | 6 | 6.06 |
| Copepod*Asperigllus*time | 10 | 5.6 | Copepod*Aspergillus+time | 7 | 9.38 | Copepod*time | 6 | 6.09 |
| Aspergillus+time | 5 | 5.68 | Copepod | 4 | 10 | Copepod+Aspergillus+time | 6 | 6.12 |
| Copepod+Aspergillus+time | 6 | 6.05 | Aspergillus | 4 | 10 | Copepod*Aspergillus | 6 | 7 |
| Parasite_single+time | 6 | 6.21 | Parasite_single | 5 | 10.3 | Copepod*Asperigllus*time | 10 | 7.03 |
| Copepod*time+Aspergillus | 7 | 7.02 | Copepod+Aspergillus | 5 | 12 | Copepod*time+Aspergillus | 7 | 7.94 |
| Copepod*Aspergillus+time | 7 | 7.27 | Copepod*Aspergillus | 6 | 12.3 | Copepod*Aspergillus+time | 7 | 8.02 |

**Table S6:** Models tested for immune-related metrics in the laboratory experiment: Only models with the “parasite” term were tested for IκB because random slope analyses supported the use of the “parasite” term as a random slope.

| **Metric** | **Treatment models** | **Parasite models** | **Parasite single models** |
| --- | --- | --- | --- |
| Amoebocyte density | - *Aspergillus* - *Aspergillus* + time - *Aspergillus* * time - Copepod - Copepod + time - Copepod * time - *Aspergillus* * Copepod - *Aspergillus* * Copepod + time - *Aspergillus* * Copepod * time - *Aspergillus* + Copepod - *Aspergillus +* Copepod + time - *Aspergillus* * time + Copepod - Copepod * time + *Aspergillus* | - Parasite - Parasite + time - Parasite * time | - Parasite_single - Parasite_single + time - Parasite_single * time |
| MMP (no random slope) |  |  |  |
| IκB (random slope = parasite) | None |  |  |
| T5A (random slope = time) | - *Aspergillus* + time - *Aspergillus* * time - Copepod + time - Copepod * time - *Aspergillus* * Copepod + time - *Aspergillus* * Copepod * time - *Aspergillus +* Copepod + time - *Aspergillus* * time + Copepod - Copepod * time + *Aspergillus* | - Parasite + time - Parasite * time | - Parasite_single + time - Parasite_single * time |

**Table S7:** Best fit random effects structures for candidate genes

| **Gene** | **Random slope** | **Random intercept** |
| --- | --- | --- |
| T5A | Time | Colony |
| IκB | ParasiteYN | Colony |
| MMP | None | Colony |

**Table S8:** AIC Table of models for amoebocyte density in the laboratory experiment (random effects = 1|colony:sample) with the best model highlighted. The model with copepod alone is better than the null by the LRT (Chi Sq. = 3.99, P= 0.0457).

| **Model** | **Degrees of freedom (K)** | **Delta AIC** | **AIC Weight** |
| --- | --- | --- | --- |
| Copepod | 5 | 0 | 0.21 |
| Copepod + Aspergillus | 6 | 1.24 | 0.12 |
| Parasite_single | 6 | 1.74 | 0.09 |
| Parasite | 5 | 1.91 | 0.08 |
| Copepod + time | 6 | 1.95 | 0.08 |
| Null | 4 | 1.99 | 0.08 |
| Copepod * Aspergillus | 7 | 3.08 | 0.05 |
| Copepod + Aspergillus + time | 7 | 3.18 | 0.04 |
| Aspergillus | 5 | 3.21 | 0.04 |
| Parasite_single + time | 7 | 3.68 | 0.03 |
| Parasite + time | 6 | 3.84 | 0.03 |
| Copepod * time | 7 | 3.93 | 0.03 |
| Copepod+Aspergillus*time | 8 | 4.09 | 0.03 |
| Copepod * Aspergillus + time | 8 | 5.03 | 0.02 |
| Copepod * time + Aspergillus | 8 | 5.15 | 0.02 |
| Aspergillus + time | 6 | 5.15 | 0.02 |
| Parasite *time | 7 | 5.65 | 0.01 |
| Aspergillus * time | 7 | 6.22 | 0.01 |
| Parasite_single *time | 9 | 6.75 | 0.01 |
| Copepod*Aspergillus*time | 11 | 9.75 | 0 |

**Table S9**: The best model of laboratory amoebocyte density includes the copepod as a predictor.

| **Random effects:** | |  |  |  |  |  |
| --- | --- | --- | --- | --- | --- | --- |
|  | **Groups** | **Name** | **Variance** | **Standard deviation** |  |  |
|  | sample | Intercept | 0.113 | 0.336 |  |  |
|  | colony | Intercept | 0.120 | 0.346 |  |  |
|  | Residual |  | 0.424 | 0.651 |  |  |
| **Fixed effects:** | |  |  |  |  |  |
|  | **Estimate** | **Standard**  **Error** | **Degrees of freedom** | **t value** | **Pr(>\|t\|)** | **95% CI** |
| Intercept | 4.54 | 0.127 | 8.08 | 35.9 | 3.40E-10 | 4.29-4.79 |
| Copepod | 0.105 | 0.0525 | 64.8 | 2.01 | 0.049 | 0.00242-0.208 |

**Fig. S3**: (a) Amoebocyte density is best predicted by copepod infection across all 9 colonies included in the analyses. (b) Amoebocyte density across all treatments (error bars are 1SE).

a)

b)


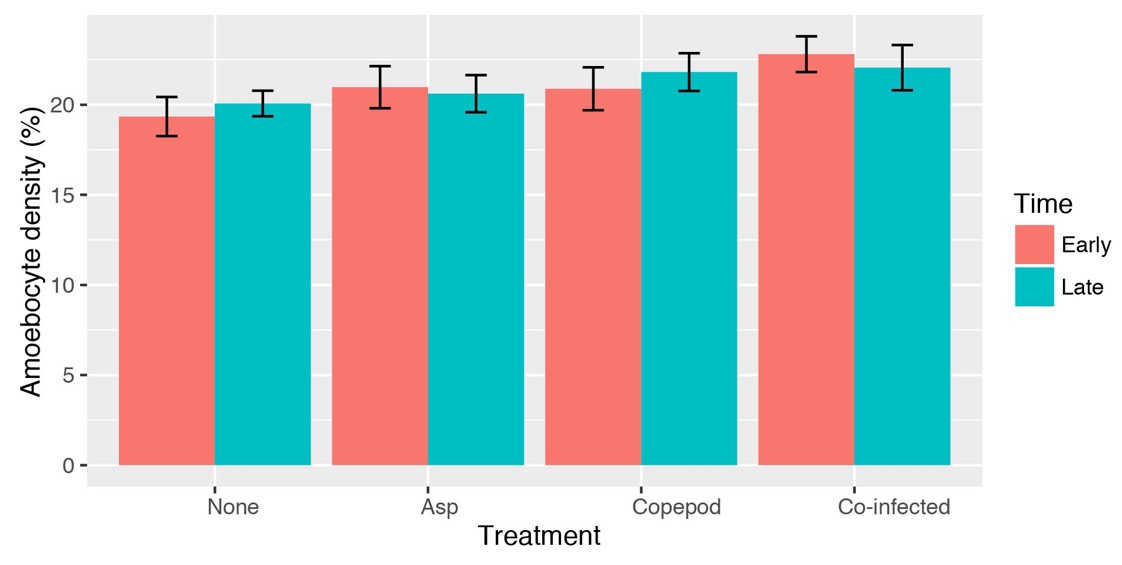


**Table S10**: AIC Table of models for T5A expression in the laboratory experiment (random effect: time| colony) with the best model highlighted. No models were tested without time because it was the best random slope. The highlighted model is better than the null model (Chi Sq.= 14.108, P= 8.64E-04) and the Parasite*time model by the LRT (Chi Sq.= 2.01, P= 0.156).

| **Model** | **Degrees of freedom** | **Delta AIC** |
| --- | --- | --- |
| Parasite*time | 8 | 0 |
| Parasite+time | 7 | 0.0112 |
| Parasite_single+time | 8 | 0.673 |
| Parasite_single*time | 10 | 2.18 |
| Copepod*Aspergillus+time | 9 | 2.65 |
| Copepod*Aspergillus*time | 12 | 5.20 |
| Copepod*time+Aspergillus | 9 | 9.17 |
| Copepod*time | 8 | 9.49 |
| Copepod+Aspergillus+time | 8 | 9.84 |
| Copepod+time | 7 | 10.0 |
| Null | 5 | 10.1 |
| Aspergillus+time | 7 | 10.6 |
| Copepod+Aspergillus+time | 9 | 11.7 |
| Aspergillus*time | 8 | 12.5 |

**Table S11**: The best model of Tachylectin 5A (T5A) included the general Parasite term (Parasite) and time.

| **Random effects:** | |  |  |  |  |  |
| --- | --- | --- | --- | --- | --- | --- |
|  | **Groups** | **Name** | **Variance** | **Std.Dev.** | **Corr** |  |
|  | colony | (Intercept) | 0.0244 | 0.156 |  |  |
|  |  | time1 | 6.65E-03 | 0.0815 | 0.3 |  |
|  | Residual |  | 0.00523 | 0.0723 |  |  |
| **Fixed effects:** | |  |  |  |  |  |
|  | **Estimate** | **Std. error** | **df** | **t value** | **Pr(>\|t\|)** | **95% CI** |
| (Intercept) | 0.401 | 0.0530 | 8.14 | 7.56 | 6.0E-05 | 0.297-0.505 |
| Parasite | 0.0369 | 0.00984 | 53 | 3.75 | 4.4E-04 | 0.0176-0.0562 |
| Time | 0.0330 | 0.0285 | 8 | 1.16 | 0.280 | -0.0228-0.0888 |

**Fig. S4**: T5A expression across all treatments and time points (error bars are 1SE).


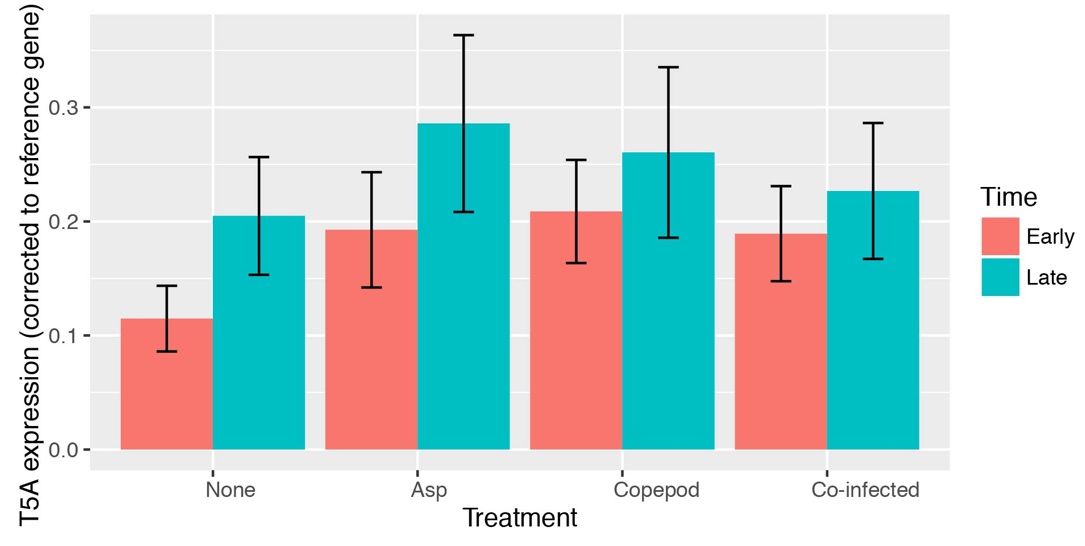


**Table S12**: AIC tables of models for IκB expression in the laboratory experiment. Two sets of models were tested with different random effects because Parasite and Parasite_single were both supported as random slopes. (a) Models with the random effect: Parasite| colony: Parasite*time is the best model by AIC, but the null model is best by the LRT (Chi Sq. = 2.01, P= 0.156). (b) Models with the random effect: Parasite_single | colony: the null model is the best model by AIC and parsimony.

(a)

| **Model** | **Degrees of freedom** | **Delta AIC** |
| --- | --- | --- |
| Parasite * time | 8 | 0 |
| Null | 5 | 1.06 |
| Parasite | 6 | 1.69 |
| Parasite + time | 7 | 1.76 |

(b)

| **Model** | **Degrees of freedom** | **Delta AIC** |
| --- | --- | --- |
| Null | 8 | 0 |
| Parasite_single *time | 13 | 1.20 |
| Parasite_single | 10 | 2.63 |
| Parasite_single +time | 11 | 2.70 |

**Table S13**: The best model of IκB gene expression by AIC (parasite *time with Parasite as random slope), supports a trend for increasing expression in the presence of a parasite during the early time point only, though the null is the best model by the LRT (P=0.07).

|  | **Estimate** | **Std. error** | **Degrees of freedom** | **t value** | **Pr(>\|t\|)** |
| --- | --- | --- | --- | --- | --- |
| **(Intercept)** | 0.261 | 0.0201 | 8 | 13.0 | 0.0000 |
| **Parasite** | 0.0178 | 0.0155 | 8 | 1.15 | 0.285 |
| **Time** | -0.0153 | 0.00696 | 52 | -2.20 | 0.0322 |
| **Parasite:time** | 0.0135 | 0.00696 | 52 | 1.94 | 0.0583 |

**Fig. S5**: IκB expression across all treatments and time points (error bars are 1SE).

**
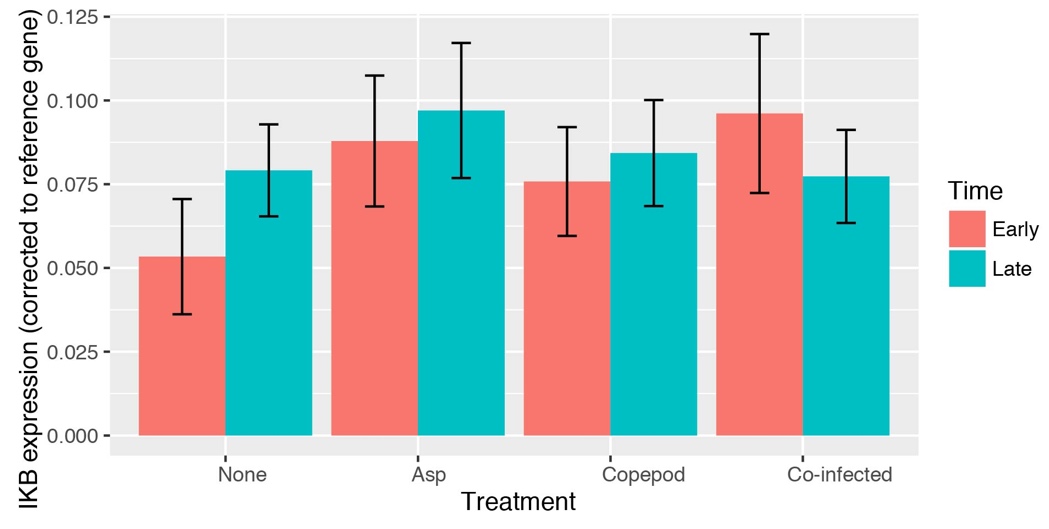
**

**Table S14**: AIC Table of models for MMP expression in the laboratory experiment (random effect: 1| colony) with the best model highlighted.

| **Mode**l | **Degrees of freedom** | **Delta AIC** |
| --- | --- | --- |
| Null | 3 | 0 |
| Copepod | 4 | 1.021 |
| Copepod * time | 6 | 1.1756 |
| Parasite * time | 6 | 1.3558 |
| Parasite | 4 | 1.5652 |
| Aspergillus | 4 | 1.90 |
| Copepod + time | 5 | 2.25 |
| Parasite + time | 5 | 2.80 |
| Copepod + Aspergillus | 5 | 2.92 |
| Copepod * time + Aspergillus | 7 | 3.07 |
| Aspergillus + time | 5 | 3.14 |
| Copepod + time + Aspergillus | 6 | 4.15 |
| Aspergillus * time | 6 | 5.14 |
| Copepod + Aspergillus * time | 7 | 6.15 |
| Copepod * Aspergillus * time | 10 | 6.67 |

**Fig. S6**: MMP expression across all treatments and time points (error bars are 1SE).


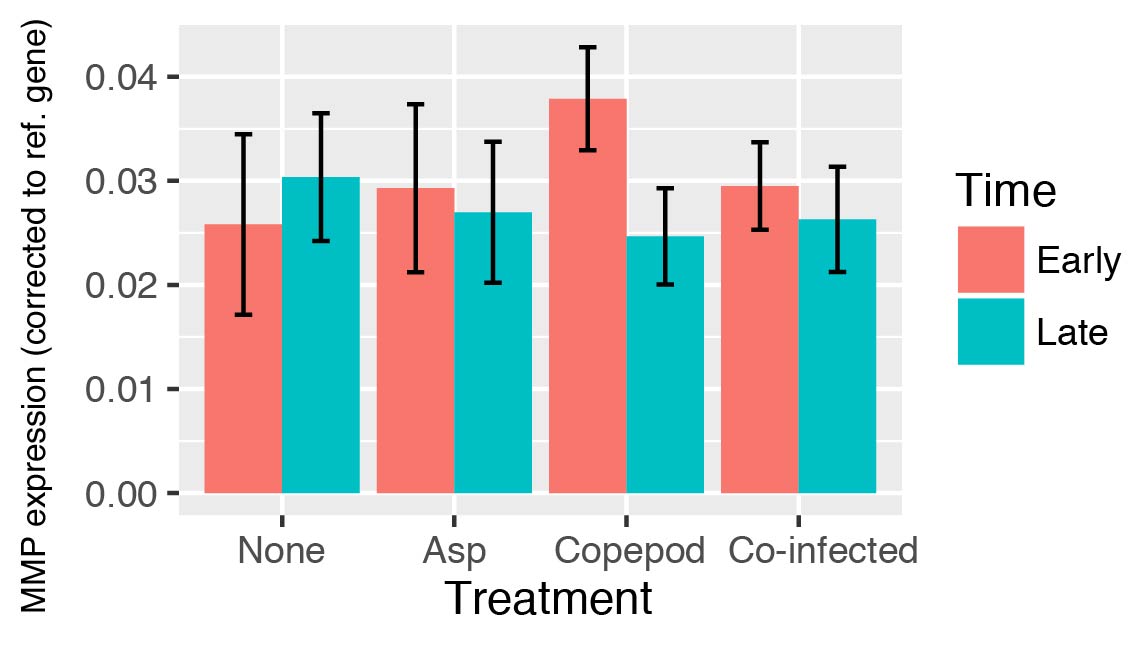


**Table S15**: Spearman’s rho correlations with Holm’s Bonferroni corrections for pairwise correlations between amoebocyte density, T5A, IκB, and MMP.

|  | **Amoebocyte density** | **T5A** | **MMP** | **IκB** |
| --- | --- | --- | --- | --- |
| **Amoebocyte density** |  |  |  |  |
| **T5A** | rho=0.411, p=0.00228 |  |  |  |
| **MMP** | rho=0.315, p=0.0289 | rho=0.394, p=0.00335 |  |  |
| **IκB** | NS  rho=0.0321, p=1.00 | NS  rho=-0.0542, p=1.00 | NS  rho=0.0583, p=1.00 |  |

**Table S16**: Copepod severity in the laboratory experiment is not predicted by time, co-infection, or their interaction.

|  | **Degrees of Freedom** | **Delta AIC** | **AIC Weight** | **Cumulative Weight** |
| --- | --- | --- | --- | --- |
| **Null** | 3 | 0 | 0.64 | 0.64 |
| **Co-infection** | 4 | 1.5 | 0.3 | 0.95 |
| **2Coinfect*Time** | 6 | 5.01 | 0.05 | 1 |

**Fig. S7:** The copepod and fungal hyphae randomly co-occur in the field. Colored bars represent observed parasite frequencies, whereas black bars represent expected frequencies under the null model for the Chi square test. 44 of the colonies had neither parasite (48.9%), 4 had only the fungus (4.44%), 34 had only the copepod (37.8%), and 8 were co-infected (8.89%).

**Table S17**: The AIC Table for amoebocyte density in the field with the best model highlighted.

| **Model** | **Degrees of freedom** | **Delta AIC** |
| --- | --- | --- |
| Copepod + site + reproductive status + coral cover | 20 | 0 |
| Fungus*copepod+ sea fan density +site +temperature +coral cover +sex | 23 | 4.38 |
| Null | 2 | 132.53 |

**Table S18**: Summary of the best model of field amoebocyte density

|  | **Estimate** | **Std. error** | **z value** | **Pr(>\|z\|)** | **Confidence interval** |
| --- | --- | --- | --- | --- | --- |
| (Intercept) | 10.0 | 0.0498 | 202 | <2.00E-16 | 9.94, 10.1 |
| Copepod infection | 0.0870 | 0.0139 | 6.28 | 3.47E-10 | 0.0597, 0.114 |
| siteCB | -0.307 | 0.0745 | -4.13 | 3.67E-05 | -0.454, -0.160 |
| siteCH | -0.277 | 0.0782 | -3.54 | 0.000407 | -0.431, -0.122 |
| siteE | -0.441 | 0.0677 | -6.51 | 7.56E-11 | -0.575, -0.307 |
| siteF | -0.107 | 0.0778 | -1.37 | 0.170 | -0.259, 0.0456 |
| siteG1 | 0.153 | 0.0747 | 2.05 | 0.0407 | 0.0055, 0.300 |
| siteG2 | -0.264 | 0.0704 | -3.75 | 0.000179 | -0.402, -0.125 |
| siteL | -0.157 | 0.0874 | -1.80 | 0.0720 | -0.329, 0.0153 |
| siteM | 0.0435 | 0.0746 | 0.583 | 0.560 | -0.101, 0.188 |
| siteML | -0.323 | 0.0724 | -4.46 | 8.17E-06 | -0.466, -0.18 |
| siteP | -0.419 | 0.0667 | -6.29 | 3.28E-10 | -0.55, -0.288 |
| siteR | -0.430 | 0.0692 | -6.22 | 5.02E-10 | -0.566, -0.295 |
| siteSC | -0.269 | 0.0706 | -3.81 | 0.000141 | -0.408, -0.129 |
| siteTC | -0.226 | 0.0752 | -3.00 | 0.00269 | -0.374, -0.0765 |
| siteTM | -0.357 | 0.0685 | -5.21 | 1.89E-07 | -0.491, -0.223 |
| Coral cover | 0.178 | 0.0448 | 3.97 | 7.18E-05 | 0.0891, 0.267 |
| Reproductive status: M vs. F | 0.00802 | 0.0175 | 0.457 | 0.647 | -0.0265, 0.0426 |
| Reproductive status: reproductive vs. not | 0.0507 | 0.0106 | 4.77 | 1.84E-06 | 0.0298, 0.0715 |

**Table S19:** The best model of amoebocyte density in a subset of the data without the deepest site supports the same predictors, but depth is not significant.

|  | **Estimate** | **Std. error** | **z value** | **Pr(>\|z\|)** |
| --- | --- | --- | --- | --- |
| (Intercept) | 9.94 | 0.145 | 68.7 | <2.00E-16 |
| Copepod infection | 0.0842 | 0.0146 | 5.77 | 7.88E-09 |
| siteCH | 0.0378 | 0.0733 | 0.516 | 0.606 |
| siteE | -0.445 | 0.200 | -2.23 | 0.0256 |
| siteF | -0.00531 | 0.158 | -0.034 | 0.973 |
| siteG1 | 0.344 | 0.103 | 3.35 | 0.000813 |
| siteG2 | -0.273 | 0.200 | -1.37 | 0.171 |
| siteL | 0.0680 | 0.102 | 0.666 | 0.505 |
| siteM | 0.227 | 0.116 | 1.95 | 0.0512 |
| siteML | -0.472 | 0.279 | -1.69 | 0.0912 |
| siteP | -0.341 | 0.155 | -2.19 | 0.0284 |
| siteR | -0.430 | 0.198 | -2.17 | 0.0298 |
| siteSC | -0.279 | 0.199 | -1.40 | 0.161 |
| siteTC | -0.242 | 0.202 | -1.19 | 0.233 |
| siteTM | -0.347 | 0.197 | -1.76 | 0.0791 |
| Coral cover | 0.190 | 0.0463 | 4.11 | 3.97E-05 |
| Depth | 0.260 | 0.150 | 1.73 | 0.0839 |
| Reproductive status: M vs. F | -0.000141 | 0.0188 | -0.007 | 0.994 |
| Reproductive status: reproductive vs. not | 0.0663 | 0.0112 | 5.91 | 3.49E-09 |

**Table S20**: The AIC Table for MFPS prevalence with the best model highlighted.

|  | **Degrees of freedom** | **Delta AIC** | **AIC Weight** |
| --- | --- | --- | --- |
| Size^2^ + temperature + coral cover | 6 | 0 | 1 |
| Coral cover + depth + temperature + size | 6 | 75.2 | 0 |
| null | 2 | 164.32 | 0 |

**Table S21**: The best model of MFPS prevalence includes size^2^ as a predictor.

| Random effects | | | | | | | | |
| --- | --- | --- | --- | --- | --- | --- | --- | --- |
|  | **Groups** | **Name** | **Variance** | | **Std.Dev** |  |  |  |
|  | site | (Intercept) | 0.290 | | 0.538 |  |  |  |
| Fixed effects: | | | | | | **95% CI** | | |
|  | **Estimate** | **Std. error** | **z value** | **Pr(>\|z\|)** | | **Left** | **Right** | |
| **(Intercept)** | -0.683 | 0.156 | -4.38 | 1.21E-05 | | -0.978 | -0.377 | |
| **size** | 2.46 | 0.209 | 11.8 | <2.00E-16 | | 2.05 | 2.87 | |
| **I(size^2)** | -1.13 | 0.154 | -7.38 | 1.57E-13 | | -1.43 | -0.833 | |
| **Temper-ature** | -0.647 | 0.292 | -2.216 | 0.0267 | | -1.22 | -0.0747 | |
| **Coral cover** | -0.396 | 0.235 | -1.69 | 0.0911 | | -0.856 | 0.0634 | |

**Table S22**: The AIC Table for MFPS severity with the best model highlighted. Site was tested separately with each predictor due to convergence errors.

| **Model** | **DF** | **Delta AIC** |
| --- | --- | --- |
| Size^2^+site | 18 | 0 |
| Size+site | 17 | 4.41 |
| Size^2^+temperature | 8 | 17.7 |
| Size+temperature+copper | 7 | 20.4 |
| Size^2^+coral cover | 8 | 21.7 |
| Size+coral cover+copper | 7 | 24.6 |
| Coral cover+site | 17 | 32.3 |
| Site | 16 | 35.1 |
| Null | 2 | 57.0 |

**Table S23**: The summary of the best model for MFPS severity includes size^2^ and site (Table S2) as predictors.

|  | **Estimate** | **Std Error** | **t value** | **Pr(>t)** | **95% CI** |
| --- | --- | --- | --- | --- | --- |
| (Intercept) | 2.52 | 0.295 | 8.54 | <2.00E-16 | 1.95, 3.10 |
| Size | 0.834 | 0.148 | 5.63 | 1.83E-08 | 0.543, 1.12 |
| Size^2^ | -0.256 | 0.121 | -2.11 | 0.0346 | -0.493, -0.0185 |
| SiteCB | -0.511 | 0.358 | -1.428 | 0.153 | -1.21, 0.191 |
| SiteCH | 0.015 | 0.334 | 0.043 | 0.965 | -0.641, 0.670 |
| SiteE | -0.709 | 0.349 | -2.03 | 0.0424 | -1.39, -0.0242 |
| SiteF | -0.767 | 0.322 | -2.38 | 0.0172 | -1.40, -0.136 |
| SiteG1 | -0.0572 | 0.344 | -0.166 | 0.868 | -0.732, 0.617 |
| SiteG2 | -0.834 | 0.390 | -2.14 | 0.0326 | -1.60, -0.069 |
| SiteL | 0.0319 | 0.307 | 0.104 | 0.917 | -0.570, 0.634 |
| SiteM | -0.708 | 0.321 | -2.21 | 0.0273 | -1.34, -0.0793 |
| SiteML | -0.615 | 0.337 | -1.83 | 0.068 | -1.27, 0.046 |
| SiteP | -0.077 | 0.358 | -0.214 | 0.830 | -0.778, 0.625 |
| SiteR | -0.299 | 0.355 | -0.841 | 0.400 | -0.994, 0.397 |
| SiteSC | -0.907 | 0.379 | -2.39 | 0.0168 | -1.65, -0.163 |
| SiteTC | -0.739 | 0.396 | -1.86 | 0.0625 | -1.52, 0.0385 |
| SiteTM | -1.05 | 0.351 | -2.99 | 0.0028 | -1.74, -0.361 |
| logSigma | -0.0008 | 0.0413 | -0.0190 | 0.985 | -0.0817, 0.0802 |

**Fig. S8:** Variability in MMP gene expression across colonies (9 colors, one colony omitted for heavy infection) across the four experimental treatments.
